# The FeatureCloud Platform for Federated Learning in Biomedicine: Unified Approach

**DOI:** 10.2196/42621

**Published:** 2023-07-12

**Authors:** Julian Matschinske, Julian Späth, Mohammad Bakhtiari, Niklas Probul, Mohammad Mahdi Kazemi Majdabadi, Reza Nasirigerdeh, Reihaneh Torkzadehmahani, Anne Hartebrodt, Balazs-Attila Orban, Sándor-József Fejér, Olga Zolotareva, Supratim Das, Linda Baumbach, Josch K Pauling, Olivera Tomašević, Béla Bihari, Marcus Bloice, Nina C Donner, Walid Fdhila, Tobias Frisch, Anne-Christin Hauschild, Dominik Heider, Andreas Holzinger, Walter Hötzendorfer, Jan Hospes, Tim Kacprowski, Markus Kastelitz, Markus List, Rudolf Mayer, Mónika Moga, Heimo Müller, Anastasia Pustozerova, Richard Röttger, Christina C Saak, Anna Saranti, Harald H H W Schmidt, Christof Tschohl, Nina K Wenke, Jan Baumbach

**Affiliations:** 1 University of Hamburg Hamburg Germany; 2 Technical University Munich Munich Germany; 3 University of Southern Denmark Odense Denmark; 4 Gnome Design SRL Sfântu Gheorghe Romania; 5 University Medical Center Hamburg-Eppendorf Hamburg Germany; 6 University of Novi Sad Novi Sad; 7 Medical University of Graz Graz Austria; 8 Concentris Research Management gGmbH Fürstenfeldbruck Germany; 9 SBA Research gGmbH Vienna Austria; 10 University Medical Center Göttingen Göttingen Germany; 11 Philipps-University of Marburg Marburg Germany; 12 Research Institute AG & Co KG Vienna Austria; 13 Technical University Braunschweig and Hannover Medical School Brunswick Germany; 14 Maastricht University Maastricht Netherlands

**Keywords:** privacy-preserving machine learning, federated learning, interactive platform, artificial intelligence, AI store, privacy-enhancing technologies, additive secret sharing

## Abstract

**Background:**

Machine learning and artificial intelligence have shown promising results in many areas and are driven by the increasing amount of available data. However, these data are often distributed across different institutions and cannot be easily shared owing to strict privacy regulations. Federated learning (FL) allows the training of distributed machine learning models without sharing sensitive data. In addition, the implementation is time-consuming and requires advanced programming skills and complex technical infrastructures.

**Objective:**

Various tools and frameworks have been developed to simplify the development of FL algorithms and provide the necessary technical infrastructure. Although there are many high-quality frameworks, most focus only on a single application case or method. To our knowledge, there are no generic frameworks, meaning that the existing solutions are restricted to a particular type of algorithm or application field. Furthermore, most of these frameworks provide an application programming interface that needs programming knowledge. There is no collection of ready-to-use FL algorithms that are extendable and allow users (eg, researchers) without programming knowledge to apply FL. A central FL platform for both FL algorithm developers and users does not exist. This study aimed to address this gap and make FL available to everyone by developing FeatureCloud, an all-in-one platform for FL in biomedicine and beyond.

**Methods:**

The FeatureCloud platform consists of 3 main components: a global frontend, a global backend, and a local controller. Our platform uses a Docker to separate the local acting components of the platform from the sensitive data systems. We evaluated our platform using 4 different algorithms on 5 data sets for both accuracy and runtime.

**Results:**

FeatureCloud removes the complexity of distributed systems for developers and end users by providing a comprehensive platform for executing multi-institutional FL analyses and implementing FL algorithms. Through its integrated artificial intelligence store, federated algorithms can easily be published and reused by the community. To secure sensitive raw data, FeatureCloud supports privacy-enhancing technologies to secure the shared local models and assures high standards in data privacy to comply with the strict General Data Protection Regulation. Our evaluation shows that applications developed in FeatureCloud can produce highly similar results compared with centralized approaches and scale well for an increasing number of participating sites.

**Conclusions:**

FeatureCloud provides a ready-to-use platform that integrates the development and execution of FL algorithms while reducing the complexity to a minimum and removing the hurdles of federated infrastructure. Thus, we believe that it has the potential to greatly increase the accessibility of privacy-preserving and distributed data analyses in biomedicine and beyond.

## Introduction

### The Problem of Scattered Data

Machine learning (ML) and artificial intelligence (AI) have increased in popularity over the last decade, leading to discoveries in various fields, including biomedicine [1-3]. The utility of ML and AI models depends on the size and quality of the available training data. However, data sources are often scattered across multiple facilities, and privacy regulations restrict data sharing, rendering large-scale, centralized ML infeasible. Particularly in biomedicine, the collection of molecular and clinical data is becoming ubiquitous with the successful applications of ML in diagnostics [4] or drug discovery [5]. Privacy concerns hinder even faster advances because of the small sample size of the individual data sets available, such as in the case of rare diseases.

### Federated Learning and Privacy-Enhancing Technologies

One way to overcome these challenges is federated learning (FL). FL allows distributed data analysis by only exchanging model parameters and local models instead of sensitive raw data [6]. Hence, analyses can benefit from considerably larger data sets and be exploited with a lower risk of revealing primary data. FL can be divided into several subcategories that address different problems in decentralized computation and differ in their requirements [7]. First, FL can be categorized according to how the data are distributed among the clients. Horizontal FL addresses the training of a model on distributed data that has the same features but different samples. Vertical FL, in contrast, trains a model for the same samples but distributed features. Second, FL is distinguished by the number of clients that participate. Training a model on decentralized data from several organizations or data silos, such as hospitals or companies, is called cross-silo FL. If model training involves thousands or millions of clients, such as mobile phones or internet of things devices, we speak of cross-device FL. A typical FL setup consists of several clients and a central aggregator. Each client updates a local model based on its local data and sends it to a central aggregator. Here, the local models are aggregated into a common global model by an aggregation function, such as federated average [6]. This global model is then broadcasted to each client again. The entire process is repeated for the iterative algorithms.

Although other techniques, such as homomorphic encryption (HE), also allow for the analysis of distributed data by enabling calculations on encrypted data directly, they are computationally expensive compared with FL. In addition, they often require drastic changes to their original ML algorithm. In contrast, FL alone cannot always fulfill strict privacy requirements [8,9]. Therefore, to improve data privacy, FL can be combined with privacy-enhancing technologies (PETs) [10], such as secure aggregation [11] or differential privacy (DP) [12,13]. A recent study demonstrated that federated algorithms could achieve comparable or identical results compared with centralized ML [14-18].

### Prior Work

Several frameworks have recently been developed to make FL available for a broader user group. Backend frameworks provide developers with methods to simplify the implementation of federated and privacy-aware algorithms [19-22]. They are limited to users with a strong background in software development or programming experience. Such skills are usually not expected from clinical experts and researchers, which considerably restricts their usability. All-in-one frameworks bring privacy-aware analyses to users without in-depth programming skills by providing a graphical user interface (GUI) [23-26]. However, most existing all-in-one frameworks are either not extendible or highly specific, focusing on a certain type of algorithm (eg, deep learning [DL] only) or application (eg, neuroimaging and genomics).

### Existing Shortcomings

Although the available frameworks demonstrate that FL is applicable and accelerates research in health care or biomedicine, the focus on 1 specific application or algorithm is also a huge restriction, especially in the collaboration of different fields. To the best of our knowledge, a generic, low-code, and open-source platform that can be driven and extended openly by the community to cover different algorithms and fields has been unavailable. However, such a platform is needed to enable FL across different applications and to make it applicable for users without technical knowledge of FL infrastructure or coding skills.

### Goal of This Work

To close this gap, we present FeatureCloud, a comprehensive platform covering all the required steps from project coordination and workflow execution for the development of algorithms for cross-silo FL [27]. It incorporates and facilitates the development and deployment of federated algorithms and alleviates the technical difficulties of end users by providing a complete and ready-to-use infrastructure. Contrary to existing programming frameworks, FeatureCloud provides a running all-in-one platform that eliminates the need for developers and users to arrange a server deployment to conduct a federated study.

## Methods

### Overview

**Figure 1:**
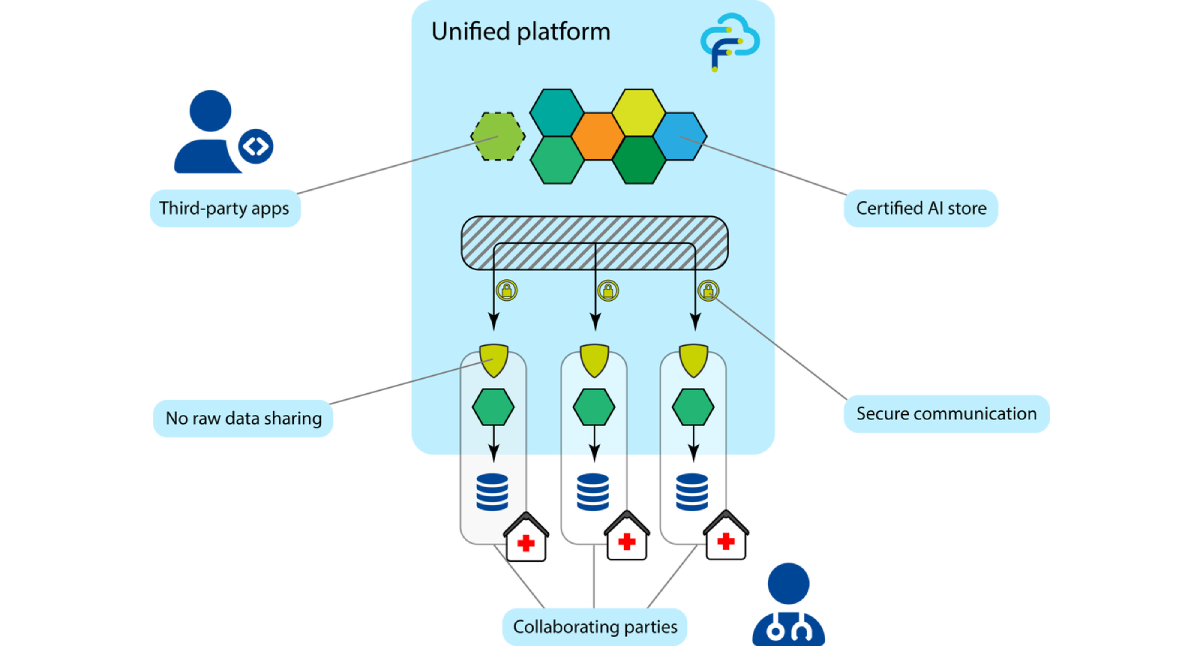
Outline of the FeatureCloud system. Medical institutions collaborate in a federated study with all primary or raw data remaining at their original location. FeatureCloud handles the distribution, execution, and communication of certified artificial intelligence (AI) applications from the FeatureCloud AI store and addresses developers and end users.

FeatureCloud was developed as a unified platform to increase the accessibility of FL for two large user groups as follows: (1) end users running FL algorithms to train ML models on distributed data sets and (2) developers implementing federated algorithms for statistics or ML that are not easily accessible in federated environments yet. As illustrated in Figure 1, the interface between developers and end users is our integrated AI store. Application developers can easily implement their own applications and publish them in the AI store, making them easily accessible to end users. Out of a broad collection of applications in the AI store, end users can assemble tailored workflows, invite collaborators, and perform FL on geographically distributed data. Therefore, FeatureCloud provides a complete infrastructure, including secure state-of-the-art communication, no raw data sharing, and several mechanisms to keep the actual data private.

### Implementation

In this section, we present our implementation of the FeatureCloud platform: its system architecture, the FeatureCloud application programming interface (API) for developers, and the FL scheme and PETs used. Furthermore, we present the FL algorithms used for the evaluation of our platform.

#### System Architecture

**Figure 2:**
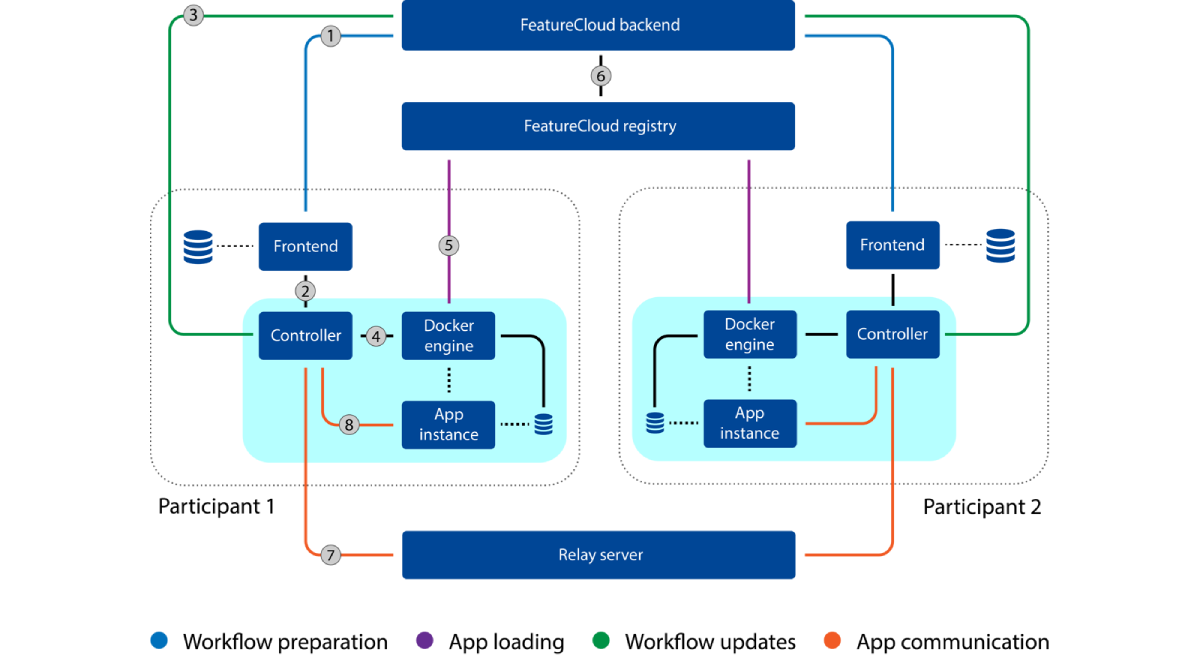
System architecture of FeatureCloud with 2 participants. The controller, frontend, Docker engine, and application instances run locally at each participant’s site. The FeatureCloud backend and Docker registry are running on FeatureCloud servers. The relay server can be run on a separate server, or participants can use a provided instance from FeatureCloud. The components are connected via transmission control protocol/IP connections (straight lines). All links are http based, except for link 7, which uses a raw socket connection. Links 1 to 3 use JSON for serialization, and links 4 to 6 use the Docker application programming interface.

FeatureCloud was developed as a system consisting of several interacting parts distributed between the participants and a central server. The central components include the backend (Python and Django), frontend (Angular), and Docker registry. The local components include the controller (Golang), the Docker engine, and the application instances (Docker images). Figure 2 shows the system components and the communication channels between them. Further details regarding their implementation and technology used can be found in Multimedia Appendix 1.

The frontend is a web application running on a web browser. It uses the FeatureCloud backend API (link 1 in Figure 2) to offer all the features of the AI store and for collaborative project management. It is also connected to the controller to allow for monitoring and handing over data for workflow runs (link 2 in Figure 2).

The controller is responsible for orchestrating the local part of the workflow execution. It receives information via the FeatureCloud backend API (link 3 in Figure 2), indicating which applications to execute next, and reports about the progress. Contrary to the relay server traffic, this traffic only contains metainformation about the execution and no data used in the algorithms themselves. It uses the Docker API (link 4 in Figure 2) to instruct the Docker engine to manage containers that serve as isolated application instances and pulls the images of the required applications for a workflow from the Docker registry (link 5 in Figure 2). When pushing new application versions, the Docker registry ensures that the user is entitled to do so by verifying their credentials through the backend (link 6 in Figure 2). In addition, the controller is an integral part of the security and privacy system of FeatureCloud. It handles local data processing and is the only part of FeatureCloud that has access to the local computer system. The controller runs in a Docker container to prevent random access to data on the system. Therefore, it only has access to selected data sets that were actively chosen by a system administrator or a user through a FeatureCloud application.

The participants of a federated workflow must also agree on a common relay server. The relay server, implemented in Go, is responsible for transmitting all traffic of the federated algorithms via a secure socket connection (link 7 in Figure 2). This central communication hub is aware of all the participants and their roles in the federated execution. It follows the required communication pattern, sending aggregated models to all the participants and local model parameters to the coordinating party only. Although FeatureCloud provides a relay server instance used by default, it is possible to use a private instance to completely shield the traffic from anyone outside the collaboration by adjusting the configuration file for the controller.

As FeatureCloud applications are a dynamic system component, partly contributed by external developers, it is necessary to isolate their implementation. This is achieved by using Docker, which ensures that they cannot access system resources other than required, especially the filesystem and network, and allows for limiting resource use, such as central processing unit or memory. They receive their input data inside a Docker volume and communicate with the *controller* through a defined API (link 8 in Figure 2). This API is the main interface between externally developed applications and the FeatureCloud system. It is http based and requires the application to act as a web server, which means that it needs to wait for the controller to query for data and cannot actively send data by itself; thus, active network access can be forbidden.

#### The FeatureCloud API for Developers

To avoid restricting end users to the current selection of applications, FeatureCloud invites external developers to implement their own federated applications and publish them in our AI store. A FeatureCloud application is a program isolated inside a Docker container that communicates with other instances using the FeatureCloud API [28]. Several templates and example applications are provided to further facilitate the implementation by directly explaining the API with code.

In addition to the AI store and the API, FeatureCloud provides tools to accelerate the development of federated applications. When developing a new federated method, application developers can directly start with the federation of the AI logic by using an existing template. To verify that the API has been implemented correctly, a simulation tool aids the developer in testing their application before publishing. Each test run specifies the number of participants, test data, and communication channels and subsequently starts the corresponding instances, simulating a real-world execution on multiple machines. During the test run, it shows logs and results for each participant and the network traffic to monitor the execution and identify bugs and potential communication bottlenecks.

After the development phase, applications can be published in the FeatureCloud AI store. Developers need to fill out a form prompting all relevant information about the application, which is displayed to the end users and used for the search and filter functions. Subsequently, they can push their Docker image into the Docker registry of the FeatureCloud platform. For end users collaborating with the developer, who explicitly enables uncertified applications, it is already usable and can be tested in a real-world scenario. For other end users, we enforce a certification process to increase the hurdle for malicious applications and maintain high privacy standards in the AI store. To this end, the developer must provide the necessary documentation and details regarding the implemented privacy mechanism. Furthermore, the application’s source code must be accessible so that the application can be exhaustively tested and vetted by the FeatureCloud team and community for possible privacy leaks. When the certification process has been successfully completed by a member of the FeatureCloud consortium according to a defined checklist (Multimedia Appendix 1), the application will be displayed in the AI store and can be used by all end users. If the certification process is unsuccessful, the developer is notified and requested to address the issues raised. Upon each update of an application, a new certification procedure is triggered.

As FeatureCloud does not impose restrictions on the types of algorithms it supports, the running environment of the federated applications is kept very general. It allows the implementation of any type of ML algorithm and an optional custom GUI for user interaction in the form of a web-based frontend. This GUI can be used to receive input parameters, indicate the current progress, or display the results. No direct internet access is granted to the applications to avoid security risks.

#### FL Scheme and PETs

FL generally involves two possibly alternating operations as follows: (1) local optimization and (2) global aggregation. In FeatureCloud, all running instances of a federated application have 1 of 2 roles (participant and coordinator) performing the respective federated operation. FeatureCloud expects precisely 1 coordinator and an arbitrary number of participants, leading to a star-based architecture. We chose this architecture over others because it mirrors the general design of a FL scheme with a central aggregator and clients with local data sets.

After the local learning operation has been completed by a participant, it sends the local parameters to the coordinator. The coordinator collects these parameters and aggregates them into a collective (global) model, which is shared with the participants again. Depending on the type of ML algorithm, these 2 operations can alternate multiple times, for example, until convergence or a predefined number of iterations has been reached (Figure S1 in Multimedia Appendix 1). For some algorithms (eg, random forest [RF] and linear regression), only 1 iteration is necessary. However, this strict separation between optimization and aggregation is not actively enforced by FeatureCloud. In many cases, aggregation can start after the first parameters have been received, thereby increasing efficiency through parallelization of the computation. During the implementation of a federated application, the distinction between the coordinator and the participant is of conceptual relevance. However, in practice, the coordinator can also obtain local data that can be used for training. Therefore, FeatureCloud allows the coordinator to simultaneously adopt the role of a participant.

Although FL improves privacy, it can still leak information to the coordinator, who can see all individual models before aggregating them. Local updates of the model based on a previously distributed global model may reveal information regarding the primary data [29]. Secure aggregation techniques can address this problem. In FeatureCloud, we integrated additive secret sharing as a mitigation method to obtain the global sum without revealing the local submodels. Application developers can use this method with minimal or no added complexity to their algorithms. More details can be found in Multimedia Appendix 1.

### Federated Algorithms

#### Comparing Federated Algorithms

As there are unique challenges for federating individual algorithms, each ML model needs to be developed independently and, therefore, needs to be based on a different underlying federation mechanism. This means that each algorithm has challenges regarding effectiveness, privacy, or scalability that need to be solved by the application developers. For the evaluation of our platform in this work, we used 4 FeatureCloud FL applications: the linear and logistic regression applications, a RF, and a DL application.

#### Federated Linear and Logistic Regression

For the implementation of the linear and logistic regression applications, the methods introduced by Nasirigerdeh et al [17] have been adapted from genome-wide association studies (GWAS) to a general ML use case. For linear regression, the local X^T^X and X^T^Y matrices are computed by each participant individually, where X is the feature matrix and Y is the label vector. Then, they are sent to the coordinator, aggregating the local matrices to the global matrices by adding them. Using these global matrices, the coordinator can calculate the beta vector through the federated method in such a way that it is identical to the beta vector calculated through the nonfederated method.

Logistic regression was implemented as an iterative approach. On the basis of the current beta vector, the local gradient and Hessian matrices of each participant are calculated and shared with the coordinator in each iteration. The coordinator aggregates the matrices again by adding them, updates the beta vector, and broadcasts it back to the participants. This process is repeated until convergence or the maximum number of iterations (prespecified for each execution) is achieved.

Internally, the scikit-learn model API has been used to implement the applications [30,31]. In the performance evaluation, we used the default scikit-learn hyperparameters for the linear regression models. For logistic regression, the penalty was set to none; the maximum number of iterations was set to 10,000; and the “lbgs” solver was used to fit the models.

#### Federated RF

We used the popular RF classifier and RF regressor as the second algorithm for our evaluation. As an ensemble algorithm, RF can be easily federated in a naive manner [32]. Our implementation trains multiple classification or regression decision trees on the local primary data of each participant. The fitted trees are then transmitted to the coordinator and merged into a global RF. To account for the different number of samples for each participant, each of them contributes a portion of the merged RF proportional to the number of samples. To achieve a similar behavior as the centralized implementation, the size of the merged RF is kept constant, meaning that an increasing number of participants decreases the number of required trees per participant. The federated computation occurs in three steps, each involving data exchange as follows: (1) participants indicate the number of samples and receive the total number of samples; (2) participants train the required number of trees, and the aggregator merges them into a global RF; and (3) participants receive the aggregated model to evaluate its performance on their data and share the results to obtain a global summary.

As the aim is not to achieve the highest possible accuracy but to compare the federated version with the nonfederated version, the hyperparameters were set to the default values of sklearn, namely, 100 decision trees, Gini impurity minimization as the splitting rule, and feature sampling equal to the square root of the features. Prepruning parameters such as maximum depth, minimum samples per node, and other constraints were not applied.

#### Federated DL

Our federated DL application is based on the federated average algorithm [6]. In the training phase, the weights and biases update is performed iteratively, where each iteration implies the parameter aggregation performed in three steps as follows: (1) the local weights and biases are computed by every participant individually and shared with the coordinator, (2) the coordinator averages the parameters and broadcasts them back to participants, and (3) the participants receive the new values of weights and biases and update the weights and biases of their model accordingly. After the final number of iterations is reached, the model performance of each participant is independently assessed using their data. The local weights and biases update is performed with the back-propagation algorithm, applied to data batches of a specified size. The neural network model architecture and training were implemented using the PyTorch library [33]. The application enables the implementation of any architecture and provides a centralized version of a PyTorch code. The application also enables federated transfer learning to be applied to a pretrained model, whose specified layers are trained in the same federated fashion.

## Results

The results comprise the unified platform and an evaluation demonstrating the technical capabilities of FeatureCloud to run different workflows. The platform consists of the open AI store, development and debugging tools, and an execution environment for federated workflows.

### Unified Platform

The unified platform (Figure 1) provides developers with an API to quickly develop privacy-enhancing FL applications. This supports a hybrid communication scheme for FL and secure aggregation (additive secret sharing). The integrated AI store is the interface between developers and end users, displaying and describing all available applications. Developers can publish (deploy) their applications in the AI store that are then available for use in federated workflows for the end users, for example, biomedical researchers. They can quickly create projects, assemble federated workflows with the applications from the AI store, invite other sites to the study, and view and download the results of each run. The interface of end users with the complicated federated architecture is reduced to only a web frontend and the FeatureCloud controller, running in the background and responsible for the local processing of sensitive data. Moreover, all applications and the entire architecture of FeatureCloud are open source, making it the first unified and open-source FL platform that considers all steps including development, deployment, and execution.

### AI Store

The integrated AI store provides an intuitive and user-friendly interface for biomedical researchers and developers. It offers a variety of applications and displays basic information about them, including short descriptions, keywords, end-user ratings, and certification status. Users can easily find applications of interest via a textual search and filter them by type (preprocessing, analysis, and evaluation) and their privacy-enhancing techniques (FL, DP, and HE). End users can review the applications and provide feedback. The application pages display a method summary, description, user reviews, developer name, and contact details to report bugs. Each application provides either a GUI or a configuration file to set the application parameters and adapt them to different contexts. This reduces technical details and makes applications user friendly for end users, independent of their background. When users add applications to their library, they can assemble them into a workflow and manage the execution with other collaborators on the FeatureCloud website without having to download any additional software.

The AI store has a broad selection of popular ML models, as listed in Table 1. The applications are categorized into preprocessing, analysis, and evaluation. Some analysis applications, such as linear regression and RF, are generic and suitable for different data types and application scenarios. These applications can be easily integrated into a federated workflow with preprocessing and evaluation applications, such as a federated standardization of the input data and a final evaluation of the trained classifier with several performance metrics. Other applications, such as the sPLINK [17] application for federated GWAS, integrate all the necessary steps of an application-specific workflow and do not require combination with other applications.

**Table 1:** Applications in the FeatureCloud artificial intelligence (AI) store^a^.

Application	Type	Description
Ada boost	Machine learning	Classification model based on boosting trees
CACS^b^ forest	Machine learning	Random forest classifying patients into their CACS
Cox PH^c^ model	Survival analysis	Survival regression based on the lifelines library
Cross-validation	Preprocessing	Local splits for a k-fold cross-validation
Deep learning	Machine learning	Deep neural networks implemented in PyTorch
Evaluation (Classification)	Evaluation	Evaluation with various classification metrics (eg, accuracy)
Evaluation (Regression)	Evaluation	Evaluation with various regression metrics (eg, mean squared error)
Evaluation (survival)	Evaluation	Evaluation of survival or time-to-event predictions
Flimma	Differential expression	Differential expression analysis based on limma-voom
Graph-guided random forest	Machine learning	Random forest classification, regression, and survival based on graphs
Kaplan-Meier estimator	Survival analysis	Survival function estimation and log-rank test
Linear regression	Machine learning	Regression model
Logistic regression	Machine learning	Classification model
Nelson-Aalen estimator	Survival analysis	Hazard function estimation and log-rank test
Normalization	Preprocessing	Standardizing input data
One-hot encoder	Preprocessing	One-hot encoding for categorical variables
Random forest	Machine learning	Classification and regression model based on decision trees
Random survival forest	Survival analysis	Survival prediction based on scikit-survival
SVD^d^	Machine learning	SVD for dimensionality reduction
sPLINK^e^	GWAS^f^	GWAS based on PLINK
Survival SVM^g^	Survival analysis	Survival prediction based on scikit-survival

^a^The growing list of applications available in the AI store covers preprocessing, analysis, and evaluation. All-in-one applications cover the entire workflow for a more specific domain and can be executed without other applications.

^b^CACS: coronary artery calcification score.

^c^PH: proportional hazard.

^d^SVD: singular value decomposition.

^e^sPLINK: secure PLINK.

^f^GWAS: genome-wide association studies.

^g^SVM: support vector machine.

### Multi-institutional Federated Workflows

FeatureCloud offers easy project management for the execution of FL workflows. In these workflows, users can select from a large variety of applications in the AI store and connect them to the entire workflow. Before collectively running a federated workflow, all collaborating sites (participants) must download and start the client-side FeatureCloud controller on their machines. It only requires Docker, which is freely available for all the major operating systems. Users also need to create an account on the FeatureCloud website, which serves as a web frontend and is used to coordinate the FeatureCloud system (refer to the *Methods* section and Multimedia Appendix 1 for details on the architecture). Each collaborative execution of applications is organized into so-called projects on the web frontend. They contain a description of the planned analysis, connect the collaborating partners by allowing invited participants to join, and show the current status of the workflow (Figure S2 in Multimedia Appendix 1).

Workflows are composed of 1 or multiple applications from the AI store that are to be executed consecutively. Each application produces intermediate results that serve as input for the consecutive application. Intermediate results are maintained on the respective machines and are not shared with other participants. The last application produces the final results, which are then shared with all the project participants. During the execution of a workflow, its progress can be monitored on the FeatureCloud website, showing the current stage, computational progress, and intermediate results from each application. Applications can provide their own user interface, allowing for user interaction if necessary and for showing specific reports. Users can monitor application logs and react in case something unexpected occurs (eg, stop and rerun the workflow with other data or a different configuration). When the last application in the workflow successfully completes its computation, the final results are automatically shared with all project participants. Intermediate results and application logs remain available on the local machines to allow for later verification. For example, the results may include a report showing the effectiveness of the trained model and the model itself. The latter can also be used outside of FeatureCloud. For example, if a project fails because a participant drops out, it can be restarted quickly after the problem has been solved. During the entire process, no programming knowledge or command-line interaction is required, making the system especially suited for medical personnel without technical education.

### Evaluation

#### Methods and Data Sets

**Figure 3:**
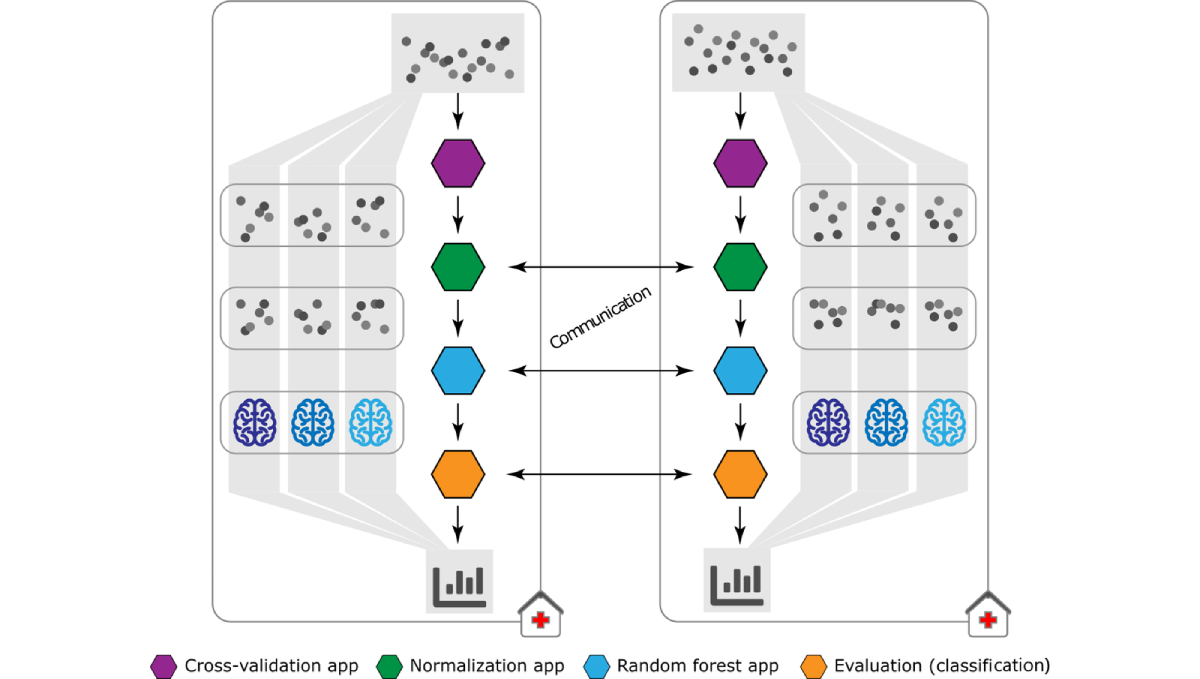
Workflow structure used for evaluation. The first application (purple—Cross-Validation) creates splits for cross-validation (CV). All following applications perform their tasks on each split individually, in a federated fashion, only transmitting model parameters. The gray dots represent intermediate training and test data. The second application (green—“Normalization”) performs normalization, and the third application (blue—“Random Forest”) trains the models, generating a global model based on the output of the normalization application. The resulting global model is evaluated in the evaluation application (orange—“Evaluation [Classification]”). The evaluation results are finally aggregated to obtain an evaluation report based on the initial CV splits.

To evaluate the practical applicability of FeatureCloud, multiple workflows operating on different data sets were created. Except for DL, each workflow consists of a cross-validation (CV) application (10-fold CV), a standardization application, a model training application, and a final evaluation application (Figure 3). For DL, we evaluated a 20% test set, as this is more common for big data to reduce the training time. Individual applications are data-type agnostic and are suitable for various applications. Classification analyses were performed on the Indian Liver Patient Dataset [34] with 579 samples and 10 features and the Cancer Genome Atlas Breast Invasive Carcinoma [35] data set with 569 samples and 20 features. For regression analyses, they were evaluated on the Diabetes [36] data set with 442 samples and 10 features and the Boston [37] house prices data set with 506 samples and 13 features, both provided by scikit-learn [30]. Finally, for DL regression, we used a large data set from the Survey of Health, Aging, and Retirement in Europe [38], with 12 questionnaire variables and the target 12-item critical assessment of protein structure prediction quality of life score. After dropping samples with “Refusal” and “Don’t know” type values in those 12 variables and nonavailable 12-item critical assessment of protein structure prediction quality of life score, we were left with 42,894 (91.79%) out of 46,733 samples. Further details regarding the network architecture are provided in Multimedia Appendix 1.

For each workflow, we split the central data set into 5 participants with uneven data distribution. Participants 1, 2 and 3, and 4 and 5 each had 10% (4289/42,894), 15% (6434/42,894), and 30% (12,868/42,894) of the samples, respectively. We used the *F*_1_-score to evaluate the classification models and the root mean squared error for the regression models, as both are common metrics used to evaluate ML models. Furthermore, we also investigated the scalability concerning runtime and network traffic for 2 to 8 participants as well as a larger number of participants and iterations.

#### Performance

**Figure 4:**
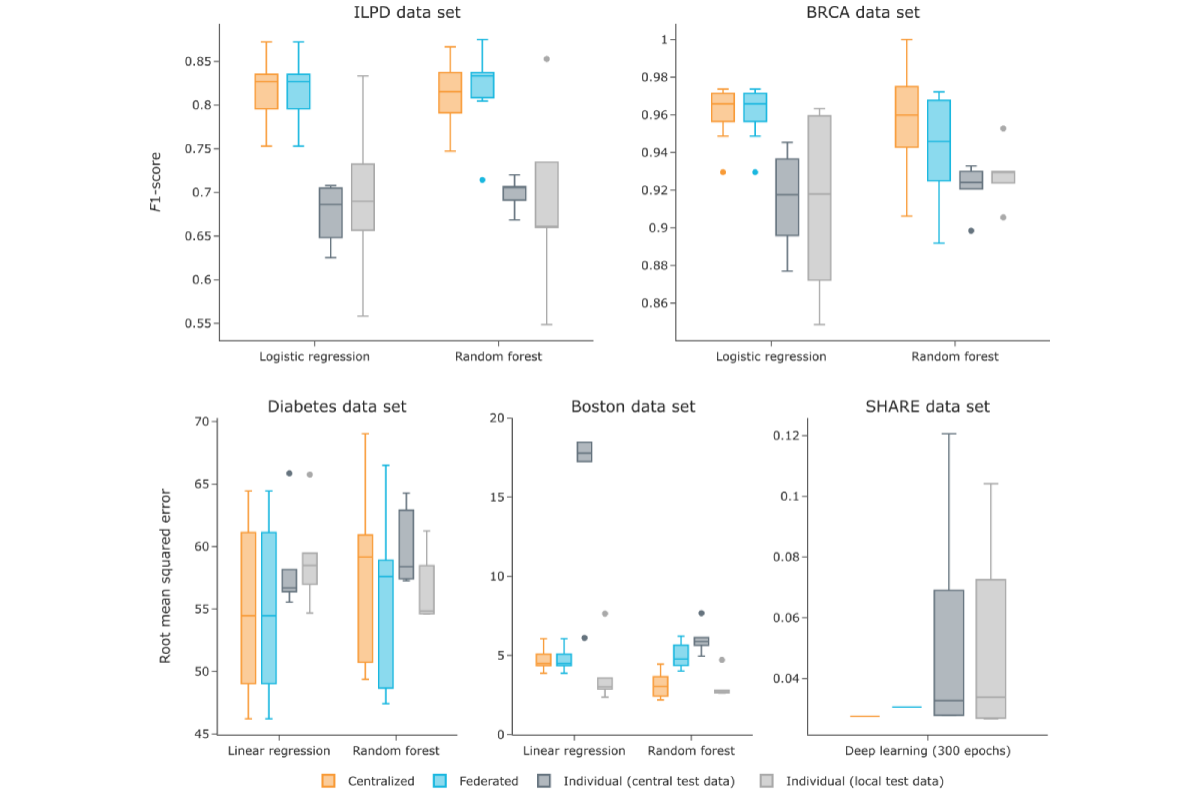
Performance evaluation of federated artificial intelligence methods. The box plots show the results of a 10-fold cross-valuation for the different classification and regression models and data sets in multiple settings. Only the deep learning model was evaluated on a test set. The centralized results are shown in orange, the corresponding federated results in blue, and the individual results obtained locally at each participant in gray. Each model was evaluated on the entire test set (dark gray) such as the centralized and federated models and on the individual (local) parts of the test set (light gray). The federated logistic and linear regressions perform in identical fashion to their centralized versions, and the federated random forest and deep learning models perform in similar fashion to their centralized versions. BRCA: Breast Invasive Carcinoma; ILDP: Indian Liver Patient Dataset; SHARE: Survey of Health, Aging and Retirement in Europe.

Previous studies have shown that FL can achieve similar performance to centralized learning in many scenarios [14,15,39]. To verify the approach used in FeatureCloud, we compared the performance of 4 federated FeatureCloud applications integrated into an ML workflow with their corresponding centralized scikit-learn [30] models. The results are shown in Figure 4. For logistic regression and linear regression, the FeatureCloud workflow achieved a performance identical to that of scikit-learn, which is consistent with the previous results of federated linear and logistic regression applications [17,40]. A similar performance was achieved for the RF regression and classification models. Owing to the simple aggregation method that combines the local trees into 1 global tree, identical results were not obtained or expected. Owing to the bootstrapping mechanism and its attached randomness, the federated RF sometimes performs slightly better than the centralized approach. As a final example, our federated DL model trained in 300 epochs produced a very close root mean squared error compared with the centralized model.

Furthermore, we compared the federated models with the individual models trained and evaluated by each participant (10-fold CV, except DL). Here, we distinguish between the central evaluation of the models on the overall test splits (central test data), identical to the test splits for the centralized and federated models, and the local evaluation of the models on the local test splits only (local test data). As shown in Figure 4, the local evaluation performance varies widely but is worse on average than the federated models. For classification, the local evaluation performed worse than the federated models. However, for the regression models, the locally evaluated models of the individual participants sometimes outperformed the centralized model. Nevertheless, compared with the central test data, it is obvious that these models did not generalize well and only performed well for the individual participants with a very small test set. This can be deceptive, as in this case, even the 10-fold CV cannot be trusted. Furthermore, our DL model evaluated on a 20% test set performs much more reliably than individual client models, which can have drastically worse results than the federated or centralized models. This highlights the effectiveness of FL, as these models use more training and test data, resulting in more generalized models. Our RF application is based on a previously published implementation [32] and confirms that our platform yields comparable results, including scenarios in which the data are neither independent nor identically distributed (nonindependent and identically distributed). It performed much more reliably than only using individual client data.

As an additional example of clinical data analysis, we evaluated the Kaplan-Meier estimator application that implements an already published approach for federated survival curves and a log-rank test for multi-institutional time-to-event analyses [18]. The application, implemented and run in FeatureCloud, produced identical results to the centralized analysis (Table S1 in Multimedia Appendix 1) on the lung cancer data set of the North Central Cancer Treatment Group [41]. Similarly, we evaluated the Flimma application for differential gene expression analysis [16] as an example of biomedical data on a subset of 152 breast cancer expressions from the Cancer Genome Atlas repository [42] with 20,536 features. Our Flimma application produced highly similar results to those of the centralized analysis (Figure S3 in Multimedia Appendix 1). These 2 examples further show that FeatureCloud has the capability of implementing and running different approaches and bringing them into a production system.

#### Runtime and Network Traffic

Multiple executions with varying numbers of clients were performed to assess the scalability of the FeatureCloud platform and the federated methods. RF and linear regression classifiers were chosen as the iterative and noniterative methods, respectively, and both were applied to the Indian Liver Patient Dataset. Both were tested with 2, 4, 6, and 8 clients and the same number of samples to ensure comparability across the executions. To investigate the impact of network bandwidth on runtime, all executions were performed on a normal and throttled internet connection with a maximum transmission of 100 kB per second.

**Figure 5:**
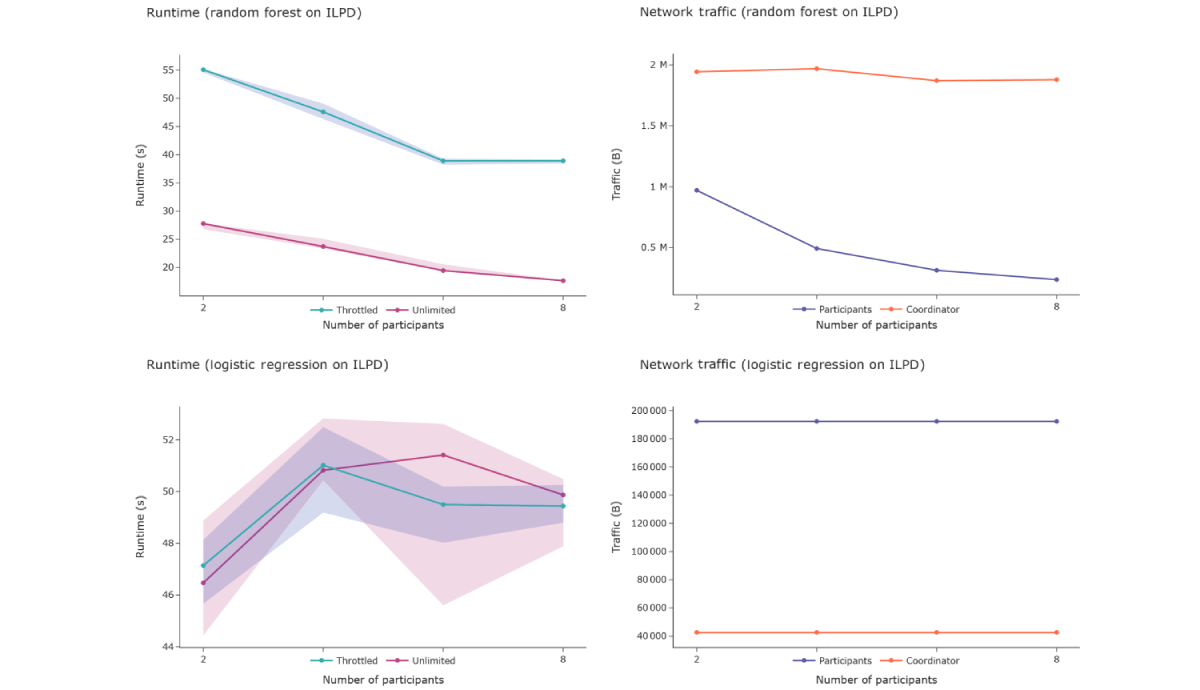
Runtime and network traffic. The left plots show runtime for unlimited and throttled connections, the right plots show network traffic for the coordinator and participants evaluated on the ILPD. The lines represent the median values measured over 10 executions. The areas show the 25% and 75% quartiles to illustrate variance across the executions. ILPD: Indian Liver Patient Dataset; s:second; B: byte; M: million.

Figure 5 shows that runtime mildly increases for logistic regression but decreases for RF. This is because the logistic regression models are of equal size for all clients, whereas the size of the RF models depends on the number of trees. In our implementation of federated RF, the global model is of a fixed size (100 trees), which means that each client contributes a portion that decreases with a higher number of participants. The throttling bandwidth significantly increases the runtime for RF but leaves the runtime for logistic regression almost unaffected. This is because the transmitted data for RF are more extensive and come in 1 chunk, whereas logistic regression requires approximately 10 iterations, each exchanging a few parameters. The centralized versions take 2 to 3 seconds to complete for both logistic regression and RF, implying that their federated versions take 10 to 20 times longer to complete.

In this setting, an increasing number of participating parties has a weak impact on the duration of the aggregation part for these methods, compared with the total runtime. The local computations occur in parallel such that an increasing number of participants does not have a huge impact. However, because the aggregation step cannot be completed before all participants send their models, the runtime of each aggregation step depends on the slowest participant, which poses a potential problem for large federations. FeatureCloud primarily focuses on being used in a tightly regulated medical research environment. Therefore, there is currently no automatic “matchmaking” in place, but all participants must join each project actively. In this context, running an analysis with data sets of >8 participants is still an uncommon scenario. To demonstrate its scalability and robustness for more sophisticated scenarios, we evaluated the FeatureCloud platform using the logistic regression application for 1, 5, 10, 15, 20, 25, and 30 clients on simulated data, with each client containing 1000 samples and 1, 5, and 10 iterations. Our analysis shows that the FeatureCloud platform is also computationally suitable for larger numbers of clients and higher numbers of iterations, confirming the results of our runtime analysis for a small number of clients (Figure S4 in Multimedia Appendix 1).

## Discussion

In this section, we summarize our main findings and provide a discussion about its comparison with prior work, its limitations, the potential for future work, and conclusions of our work.

### Summary of Results

In this study, we presented the FeatureCloud platform, a comprehensive platform for the application and development of privacy-preserving FL workflows in biomedicine and beyond. Through its high generalization, it allows the application of various ML workflows to a variety of data types. In addition, it offers prebuilt solutions for common-use cases in the form of applications in the AI store or application templates for developers. The concept of freely composing applications in a workflow is challenging because of the need for a standard data format, which is not always available and can reduce flexibility. The same applies to the initial data, which need to be provided in a form that is processable and understandable by the desired application. As FL adaptation is still in its early stages, it is necessary to understand which functionality and types of data will be used, which ML techniques prove to be most prevalent in federated settings, and which challenges arise when using the platform. Therefore, several assumptions can be made in advance.

### Comparison With Prior Work

One main goal of FeatureCloud was to keep the platform as flexible and extensible as possible, to align new functionality closely to the demand of its users. The possibility of integrating additional PETs, such as DP or additive secret sharing, on the application layer of the API demonstrates the versatility of this approach. Although the current implementation of additive secret sharing has a quadratic increase in network traffic, it shows that flexible communication can be achieved through asymmetrical encryption and can serve as a blueprint for similar scenarios and future developments.

The prediction performance of our FL workflows is consistent with the current research, with some performing equally well compared with the central implementations (linear and logistic regression and normalization) or highly similar (RF). Computational and communication overheads are acceptable for an ordinary FL. In our opinion, it plays a smaller role than the additional overhead related to human-to-human coordination of federated projects. We demonstrated that the currently available applications and the platform scale well for up to 8 participants.

The main novelty, in contrast to prior work, is the high flexibility of the AI store, ranging from prebuilt task-centered applications, such as GWAS, to generic method-centered applications, such as RF. Therefore, we address a broad spectrum of end users and developers. Less experienced users without deeper methodological or statistical knowledge benefit from the ease of use of a task-centered application. Advanced users can tailor the workflow to their needs. In contrast, application developers can use our API to develop FL applications that can be easily deployed into the AI store and reach a broad user base. They are incentivized to build their applications to be compatible with existing ones (eg, a new AI method that processes data preprocessed by an existing normalization application) to maximize their utility. Thus, the FeatureCloud AI store aims to become an ecosystem for FL, driving collaborative research.

### Limitations

In addition to the huge potential of FeatureCloud, some issues still need to be addressed. Our secure aggregation approach, directly implemented into the developer API, only applies to ≥3 participants. Its application on workflows with only 2 participants would allow the coordinator to reveal the local parameters of the other participant and therefore has no benefit. In addition, as it is currently implemented, our additive secret-sharing approach only supports addition and multiplication and is, therefore, not applicable to more complex types of calculations. Although the open AI store accelerated the development and deployment of FL applications and workflows, it is the responsibility of the application developers to provide proof that their implementations provide accurate results. FeatureCloud certifies applications that provide a reasonable amount of privacy and security measures but cannot check the prediction quality of every application. However, through its open-source design, the community can exchange experiences, provide feedback, and enhance applications and algorithms to keep them up to date with the current state of the art.

### Future Work

The generic and extendable design of FeatureCloud makes it highly interesting for future studies. FeatureCloud envisions being driven by an emerging community whose features are closely aligned to their needs. As FeatureCloud is entirely open source, it can be quickly maintained and extended and it can accelerate the development, deployment, and execution of privacy-preserving FL workflows in biomedicine and other areas. FeatureCloud applications can be developed by anyone using the developer API and easy-to-start templates. One part could focus on integrating more PETs into the API for the application developers to ease their use and increase adoption in federated algorithms. Although FeatureCloud already integrates an additive secret-sharing scheme, there are many more PETs, such as DP or HE schemes, that can be implemented. Other potential enhancements could focus on nonlinear workflows, the integration of the AIMe registry [43] into the certification process of FeatureCloud applications, and reducing Docker dependency by also supporting other secure containerization systems such as Singularity [44]. To address the problem of data harmonization and preprocessing of different formats at different sites, it may be useful to add a federated database with a common ontology to the FeatureCloud controller [45]. Through this, the problem of different data formats between sites is solved, as the input data for workflows can be directly created from the database. Integrating local data into this database can be performed using predefined Extract-Transform-Load scripts for the most common data formats and standards.

### Conclusions

In conclusion, FeatureCloud provides an all-in-one platform for privacy-preserving FL. In contrast to other FL frameworks, FeatureCloud considers every aspect of FL from development and deployment to the execution and project planning of federated analyses. Furthermore, it is highly generic to support all types of algorithms and is not restricted to only DL or a certain application. Thus, we believe that it has a huge potential to accelerate the development of FL workflows and the application of federated analyses in biomedicine.

## Appendix

Multimedia Appendix 1 Additional information containing figures and descriptions related to software architecture and implementation.

## Abbreviations

AI: artificial intelligence
API: application programming interface
CV: cross-validation
DL: deep learning
DP: differential privacy
FL: federated learning
GUI: graphical user interface
GWAS: genome-wide association studies
HE: homomorphic encryption
ML: machine learning
PET: privacy-enhancing technology
RF: random forest

## Data Availability

The Survey of Health, Aging and Retirement in Europe (SHARE) data are distributed by SHARE-European Research Infrastructure Consortium (ERIC) to registered users through the SHARE Research Data Center. We used only data from the 8 waves [38]. Except for the SHARE data, all our data sets, including the Indian Liver Patient Dataset [34], Breast Invasive Carcinoma data set [35], Boston data set [37], and Diabetes data set [36], and scripts used for our evaluation results are available in our GitHub repository [47]. To increase interpretation and reproducibility, we followed the minimum information about clinical artificial intelligence modeling (ML-CLAIM) reporting standard (Norgeot et al [48]). The filled-out ML-CLAIM clinical checklist is also available in our GitHub repository.

## References

[1] Mamoshina P, Vieira A, Putin E, Zhavoronkov A (2016). Applications of deep learning in biomedicine. Mol Pharm.

[2] Yu KH, Beam AL, Kohane IS (2018). Artificial intelligence in healthcare. Nat Biomed Eng.

[3] Malle B, Giuliani N, Kieseberg P, Holzinger A (2017). The more the merrier - federated learning from local sphere recommendations. Proceedings of the 1st International Cross-Domain Conference on Machine Learning and Knowledge Extraction.

[4] Esteva A, Kuprel B, Novoa RA, Ko J, Swetter SM, Blau HM, Thrun S (2017). Corrigendum: dermatologist-level classification of skin cancer with deep neural networks. Nature.

[5] Chan HC, Shan H, Dahoun T, Vogel H, Yuan S (2019). Advancing drug discovery via artificial intelligence. Trends Pharmacol Sci.

[6] McMahan HB, Moore E, Ramage D, Hampson S, Arcas BA (2017). Communication-efficient learning of deep networks from decentralized data. Proceedings of the 20th International Conference on Artificial Intelligence and Statistics.

[7] Kairouz P, McMahan HB, Avent B, Bellet A, Bennis M, Nitin Bhagoji A, Bonawitz K, Charles Z, Cormode G, Cummings R, D’Oliveira RG, Eichner H, El Rouayheb SE, Evans D, Gardner J, Garrett Z, Gascón A, Ghazi B, Gibbons PB, Gruteser M, Harchaoui Z, He C, He L, Huo Z, Hutchinson B, Hsu J, Jaggi M, Javidi T, Joshi G, Khodak M, Konecný J, Korolova A, Koushanfar F, Koyejo S, Lepoint T, Liu Y, Mittal P, Mohri M, Nock R, Özgür A, Pagh R, Qi H, Ramage D, Raskar R, Raykova M, Song D, Song W, Stich SU, Sun Z, Suresh AT, Tramèr F, Vepakomma P, Wang J, Xiong L, Xu Z, Yang Q, Yu FX, Yu H, Zhao S (2021). Advances and open problems in federated learning. Found Trends Mach Learn.

[8] Tomsett R, Chan KS, Chakraborty S (2019). Model poisoning attacks against distributed machine learning systems. Proceedings of the 2019 SPIE Defense and Commercial Sensing.

[9] Usynin D, Ziller A, Makowski M, Braren R, Rueckert D, Glocker B, Kaissis G, Passerat-Palmbach J (2021). Adversarial interference and its mitigations in privacy-preserving collaborative machine learning. Nat Mach Intell.

[10] Acar A, Aksu H, Uluagac AS, Conti M (2019). A survey on homomorphic encryption schemes: theory and implementation. ACM Comput Surv.

[11] Bonawitz KA, Ivanov V, Kreuter B, Marcedone A, McMahan HB, Patel S, Ramage D, Segal A, Seth K (2017). Practical secure aggregation for privacy-preserving machine learning. Proceedings of the 2017 ACM SIGSAC Conference on Computer and Communications Security.

[12] Dwork C, McSherry F, Nissim K, Smith A (2006). Calibrating noise to sensitivity in private data analysis. Proceedings of the 3rd Theory of Cryptography Conference.

[13] Dwork C, Roth A (2014). The algorithmic foundations of differential privacy. Found Trends Theor Comput Sci.

[14] Nilsson A, Smith S, Ulm G, Gustavsson E, Jirstrand M (2018). A performance evaluation of federated learning algorithms. Proceedings of the 2nd Workshop on Distributed Infrastructures for Deep Learning.

[15] Lee GH, Shin SY (2020). Federated learning on clinical benchmark data: performance assessment. J Med Internet Res.

[16] Zolotareva O, Nasirigerdeh R, Matschinske J, Torkzadehmahani R, Bakhtiari M, Frisch T, Späth J, Blumenthal DB, Abbasinejad A, Tieri P, Kaissis G, Rückert D, Wenke NK, List M, Baumbach J (2021). Flimma: a federated and privacy-aware tool for differential gene expression analysis. Genome Biol.

[17] Nasirigerdeh R, Torkzadehmahani R, Matschinske J, Frisch T, List M, Späth J, Weiss S, Völker U, Pitkänen E, Heider D, Wenke NK, Kaissis G, Rueckert D, Kacprowski T, Baumbach J (2022). sPLINK: a hybrid federated tool as a robust alternative to meta-analysis in genome-wide association studies. Genome Biol.

[18] Späth J, Matschinske J, Kamanu FK, Murphy SA, Zolotareva O, Bakhtiari M, Antman EM, Loscalzo J, Brauneck A, Schmalhorst L, Buchholtz G, Baumbach J (2022). Privacy-aware multi-institutional time-to-event studies. PLOS Digit Health.

[19] Ryffel T, Trask A, Dahl M, Wagner B, Mancuso J, Rueckert D, Passerat-Palmbach J A generic framework for privacy preserving deep learning. arXiv.

[20] Konczyk J (2019). Federated Learning with TensorFlow.

[21] Train on the edge with federated learning. XayNet.

[22] Yang  Liu, Tao  Fan, Tianjian  Chen, Qian  Xu, Qiang  Yang (2021). An industrial grade federated learning framework. The Journal of Machine Learning Research.

[23] Gazula H, Kelly R, Romero J, Verner E, Baker BT, Silva RF, Imtiaz H, Saha DK, Raja R, Turner JA, Sarwate AD, Plis SM, Calhoun VD (2020). COINSTAC: collaborative informatics and neuroimaging suite toolkit for anonymous computation. J Open Source Softw.

[24] Silva S, Altmann A, Gutman B, Lorenzi M (2020). Fed-BioMed: a general open-source frontend framework for federated learning in healthcare. Proceedings of the 2nd MICCAI Workshop on Domain Adaptation and Representation Transfer, and Distributed and Collaborative Learning.

[25] Owkin.

[26] Melloddy.

[27] FeatureCloud - Privacy-Preserving AI.

[28] FeatureCloud AI developer API (1.1.0). FeatureCloud AI.

[29] Lyu L, Yu H, Yang Q Threats to federated learning: a survey. arXiv.

[30] Pedregosa F, Varoquaux G, Gramfort A, Michel V, Thirion B, Grisel O, Blondel M, Prettenhofer P, Weiss R, Dubourg V, Vanderplas J, Passos A, Cournapeau D, Brucher M, Perrot M, Duchesnay E (2011). Scikit-learn: machine learning in Python. J Mach Learn Res.

[31] Buitinck L, Louppe G, Blondel M, Pedregosa F, Mueller A, Grisel O, Niculae V, Prettenhofer P, Gramfort A, Grobler J, Layton R, Vanderplas J, Joly A, Holt B, Varoquaux G (2013). API design for machine learning software: experiences from the scikit-learn project. Proceedings of the 2013 European Conference on Machine Learning and Principles and Practice of Knowledge Discovery in Databases.

[32] Hauschild AC, Lemanczyk M, Matschinske J, Frisch T, Zolotareva O, Holzinger A, Baumbach J, Heider D (2022). Federated random forests can improve local performance of predictive models for various healthcare applications. Bioinformatics.

[33] Paszke A, Gross S, Massa F, Lerer A, Bradbury J, Chanan G, Killeen T, Lin Z, Gimelshein N, Antiga L, Desmaison A, Kopf A, Yang E, DeVito Z, Raison M, Tejani A, Chilamkurthy S, Steiner B, Fang L, Bai J, Chintala S (2019). PyTorch: an imperative style, high-performance deep learning library. Proceedings of the 32nd Conference on Neural Information Processing Systems.

[34] UC Irvine Machine Learning Repository.

[35] Street WN, Wolberg WH, Mangasarian OL (1993). Nuclear feature extraction for breast tumor diagnosis. Proceedings of the 1993 IS&T/SPIE'S Symposium on Symposium on Electronic Imaging: Science and Technology.

[36] Efron B, Hastie T, Johnstone I, Tibshirani R (2004). Least angle regression. Ann Stat.

[37] Harrison Jr D, Rubinfeld DL (1978). Hedonic housing prices and the demand for clean air. J Environ Econ Manage.

[38] Börsch-Supan A (2022). Survey of Health, Ageing and Retirement in Europe (SHARE) Wave 8. COVID-19 Survey 1. Release version: 8.0.0. Survey of Health, Ageing and Retirement in Europe (SHARE).

[39] Sheller MJ, Edwards B, Reina GA, Martin J, Pati S, Kotrotsou A, Milchenko M, Xu W, Marcus D, Colen RR, Bakas S (2020). Federated learning in medicine: facilitating multi-institutional collaborations without sharing patient data. Sci Rep.

[40] McMahan B, Ramage D (2017). Federated learning: collaborative machine learning without centralized training data. Google Research.

[41] Loprinzi CL, Laurie JA, Wieand HS, Krook JE, Novotny PJ, Kugler JW, Bartel J, Law M, Bateman M, Klatt NE (1994). Prospective evaluation of prognostic variables from patient-completed questionnaires. north central cancer treatment group. J Clin Oncol.

[42] Liu J, Lichtenberg T, Hoadley KA, Poisson LM, Lazar AJ, Cherniack AD, Kovatich AJ, Benz CC, Levine DA, Lee AV, Omberg L, Wolf DM, Shriver CD, Thorsson V, Hu H, Cancer Genome Atlas Research Network (2018). An integrated TCGA pan-cancer clinical data resource to drive high-quality survival outcome analytics. Cell.

[43] Matschinske J, Alcaraz N, Benis A, Golebiewski M, Grimm DG, Heumos L, Kacprowski T, Lazareva O, List M, Louadi Z, Pauling JK, Pfeifer N, Röttger R, Schwämmle V, Sturm G, Traverso A, Van Steen K, de Freitas MV, Villalba Silva GC, Wee L, Wenke NK, Zanin M, Zolotareva O, Baumbach J, Blumenthal DB (2021). The AIMe registry for artificial intelligence in biomedical research. Nat Methods.

[44] Kurtzer GM, Sochat V, Bauer MW (2017). Singularity: scientific containers for mobility of compute. PLoS One.

[45] Sheth AP, Larson JA (1990). Federated database systems for managing distributed, heterogeneous, and autonomous databases. ACM Comput Surv.

[46] Survey of Health, Aging and Retirement in Europe.

[47] Matschinske J, Späth J Evaluation - FeatureCloud. GitHub.

[48] Norgeot B, Quer G, Beaulieu-Jones BK, Torkamani A, Dias R, Gianfrancesco M, Arnaout R, Kohane IS, Saria S, Topol E, Obermeyer Z, Yu B, Butte AJ (2020). Minimum information about clinical artificial intelligence modeling: the MI-CLAIM checklist. Nat Med.

