# Supplementary material for: The FeatureCloud Platform for Federated Learning in Biomedicine: Unified Approach
Source: J Med Internet Res. 2023 Jul 12;25:e42621. doi: 10.2196/42621 (PMC10372562; doi:10.2196/42621)
Supplement: Multimedia Appendix 1 [file jmir_v25i1e42621_app1.docx]

**The FeatureCloud Platform for Federated Learning in Biomedicine: A Unified Approach**

Supplementary Material


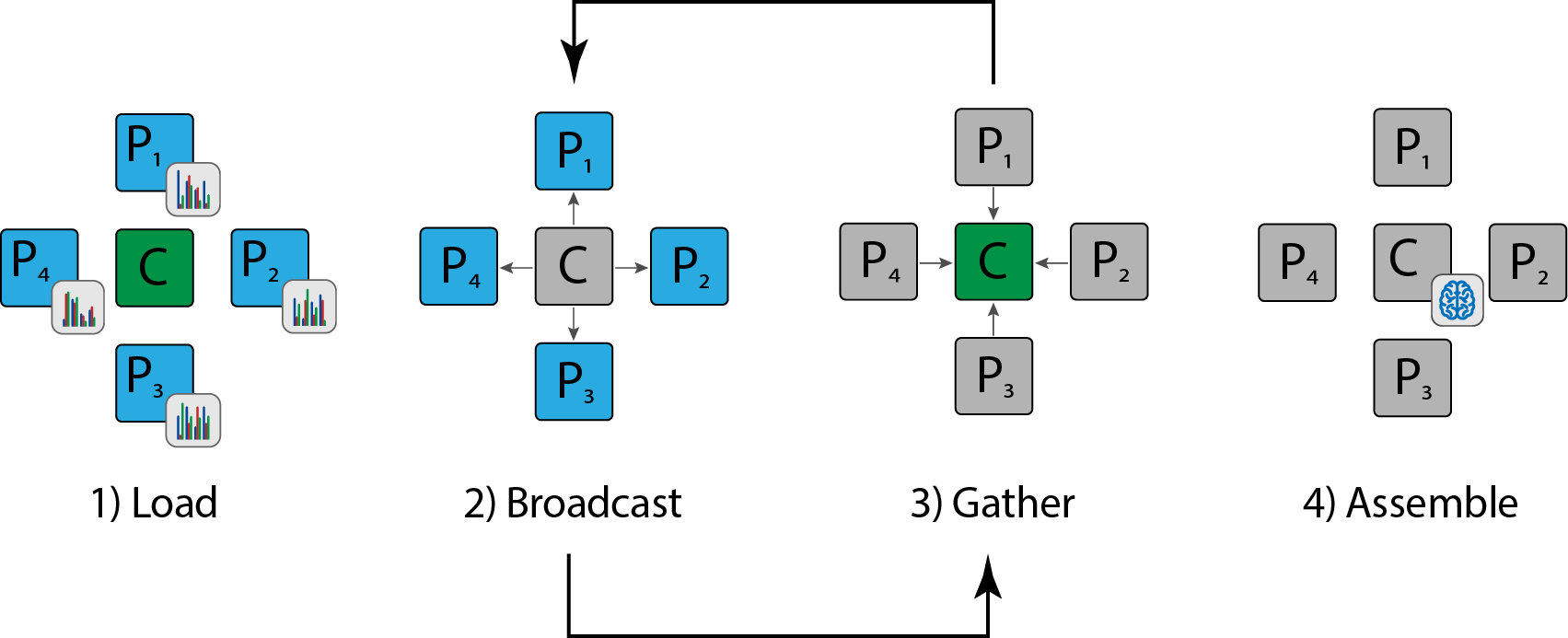


**Figure S1. Four stages of federated execution in FeatureCloud.** The four main stages are 1) local data loading, 2) broadcasting a global model, 3) gathering local models, 4) assembling results. Stages 2 and 3 can be repeated depending on the executed algorithm. ‘C’ and ‘P’ stand for coordinator and participant, respectively. This figure was inspired by [(Rieke et al., 2020)](https://paperpile.com/c/mn3PQU/p5HY).


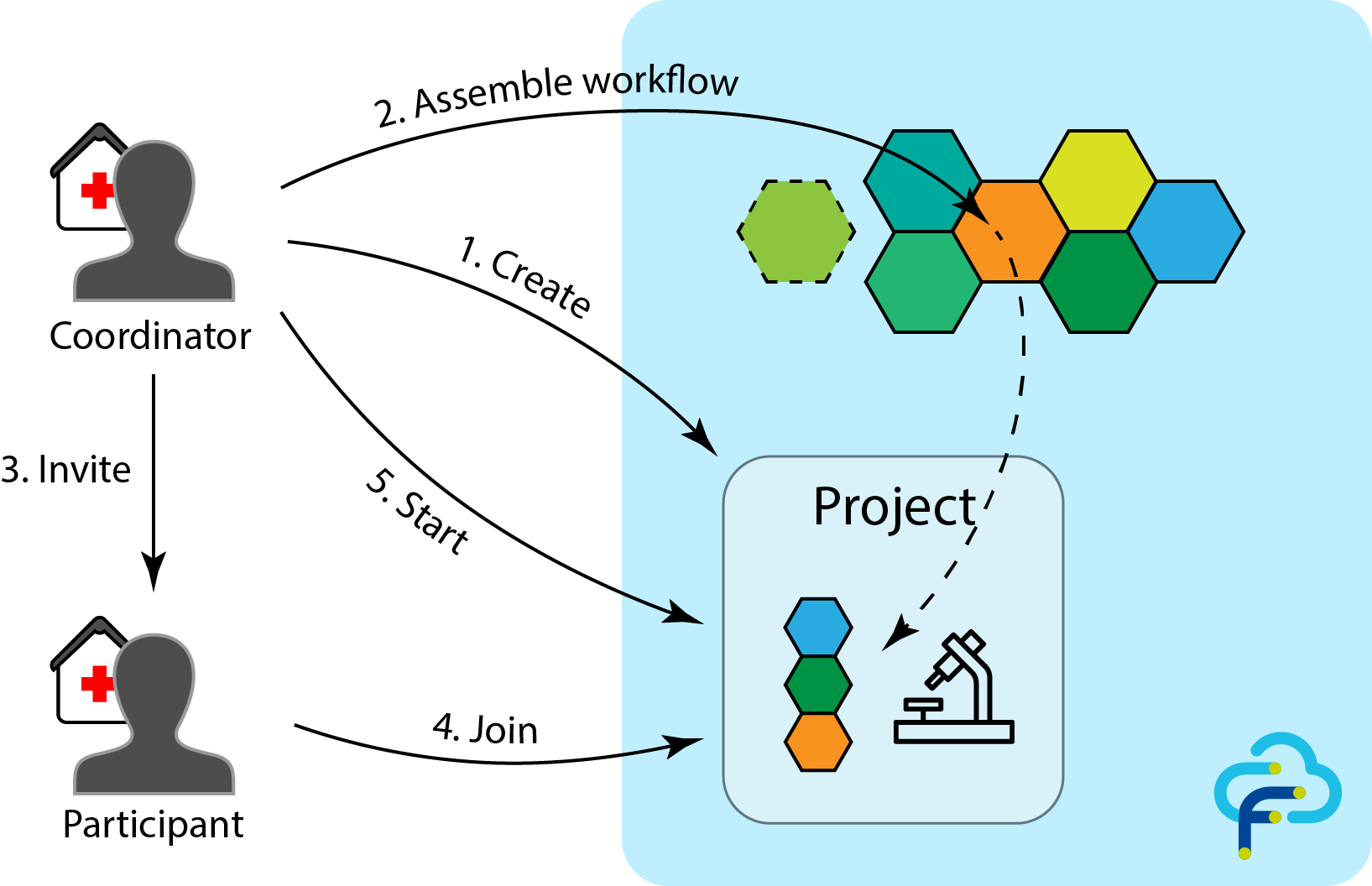


**Figure S2. Creating and setting up a federated project.** One of the partners takes the role of the coordinator and creates a new project on the website (1). After that, the coordinator defines the workflow by adding the apps to the project’s workflow (2). Then, the other partners are invited by sending a randomly generated token to each of them (3), which is unique and allows for joining the project (4). When all partners have joined, the coordinator triggers the execution on the FeatureCloud website, and the workflow runs (5). During workflow execution, active interaction with the end-user can be required, depending on the apps.


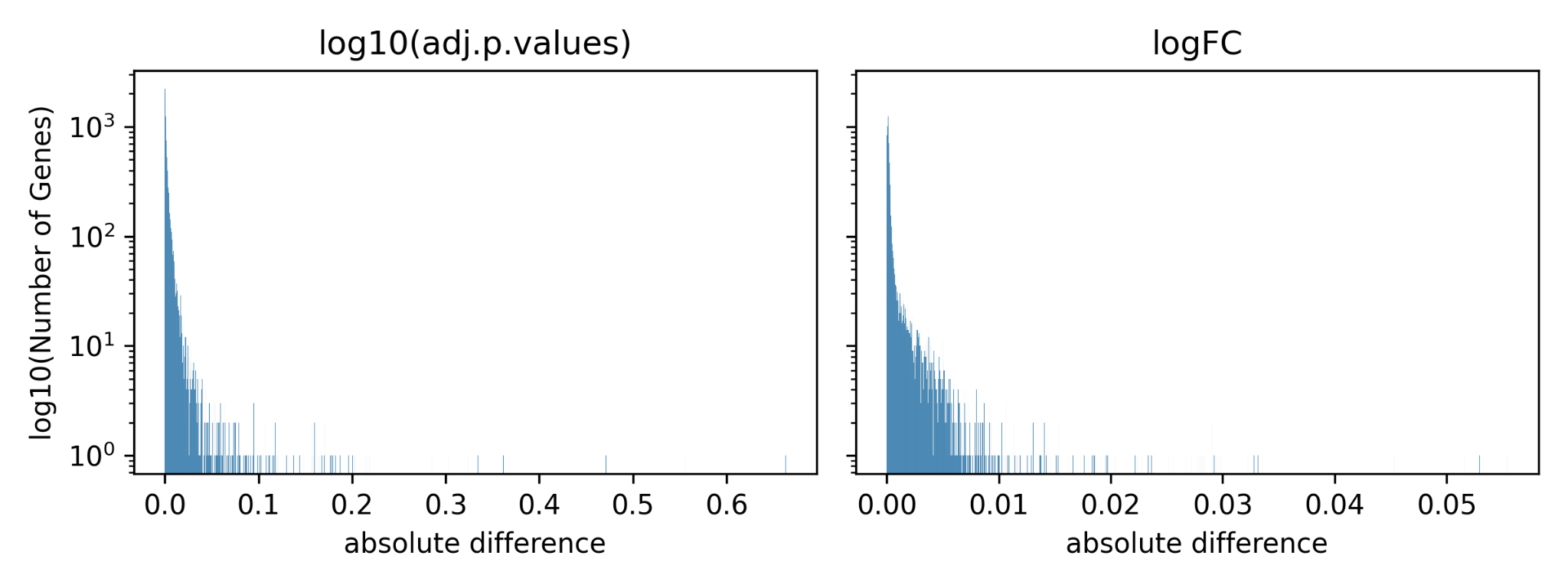


**Figure S3. Difference between the federated Flimma app and centralized limma-voom.** We compared the federated Flimma app with 5 clients with the centralized limma-voom pipeline. The absolute difference between the p-values and the logFC is very low, except for some outliers. The spearman correlation of both p-values and logFC is higher larger than 0.99999. The results are similar to the ones published in the original Flimma publication[(Zolotareva et al. 2021)](https://paperpile.com/c/mn3PQU/g4z7).


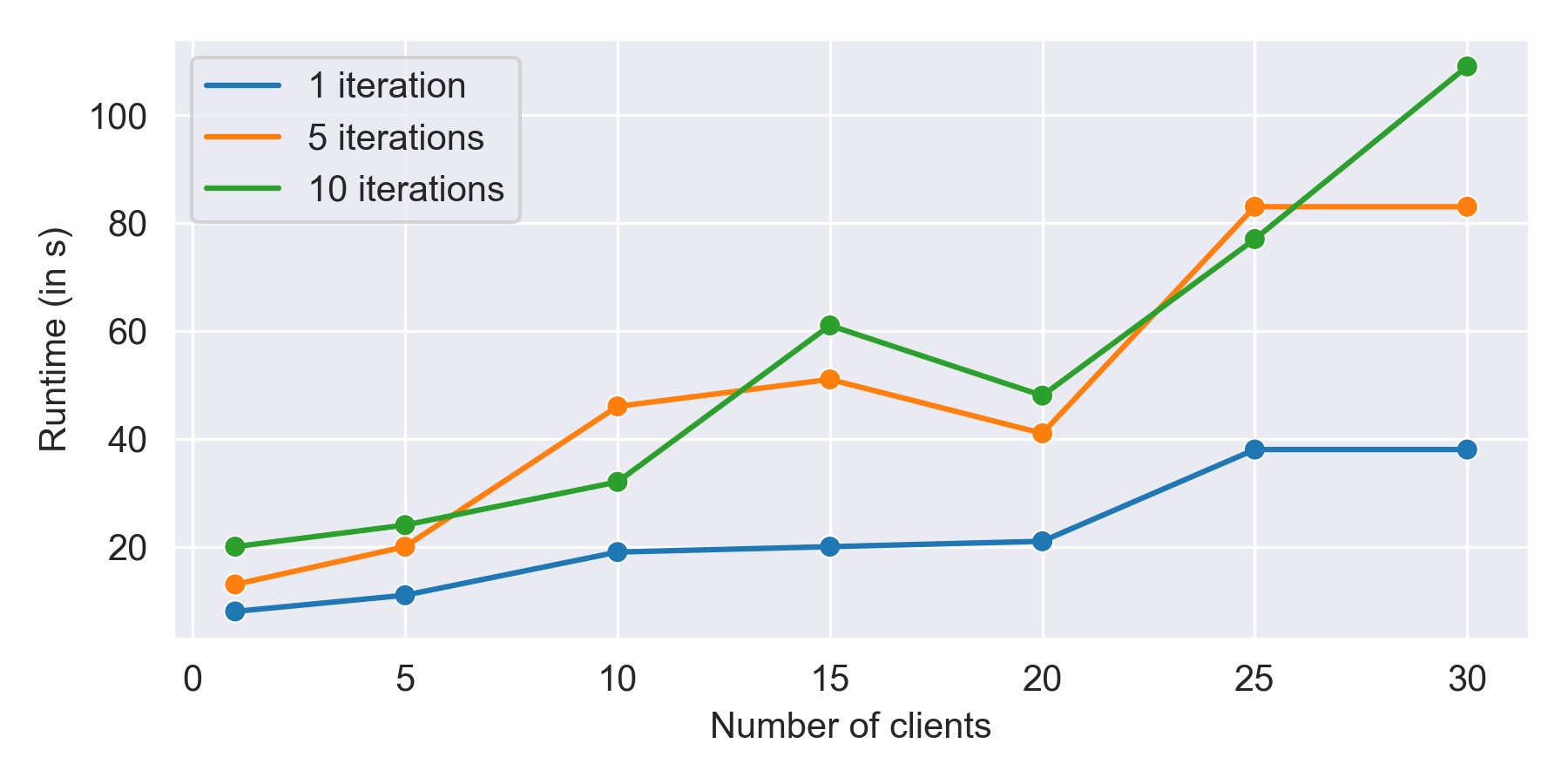


**Figure S4. Different scenarios using the logistic regression app in FeatureCloud.** We measured the runtime of the logistic regression app on artificial data (generated by sci-kit-learn’s make_classification method) for different numbers of clients (x-axis, 1000 samples per client) and various numbers of iterations (1, 5, and 10). Adding more clients and having more iterations at most increases the random linearly. Analyses were performed on a Macbook Pro M1 (2021) using the testing environment integrated into FeatureCloud.

**Table S1.** Comparison of the log-rank test between the federated Kaplan-Meier Estimator app and the logrank test provided by the python lifelines package.

|  | **Test Statistic** | **p-value** |
| --- | --- | --- |
| **Central** | 10.326742 | 0.001311 |
| **Federated (5 clients)** | 10.326742 | 0.001311 |

# A. Software architecture

This section contains information about technology, software architecture and implementation details for each of the integral FeatureCloud system components.

## Controller

The *Controller* needs to be able to handle large amounts of data and asynchronous tasks as well as keep up multiple socket connections and support HTTP-based and raw socket-based traffic. For this reason, this component has been implemented in Go (aka Golang), a native programming language developed for server applications. It allows for lightweight co-routines which are used to monitor app containers and regularly query for updates from the global backend.

*
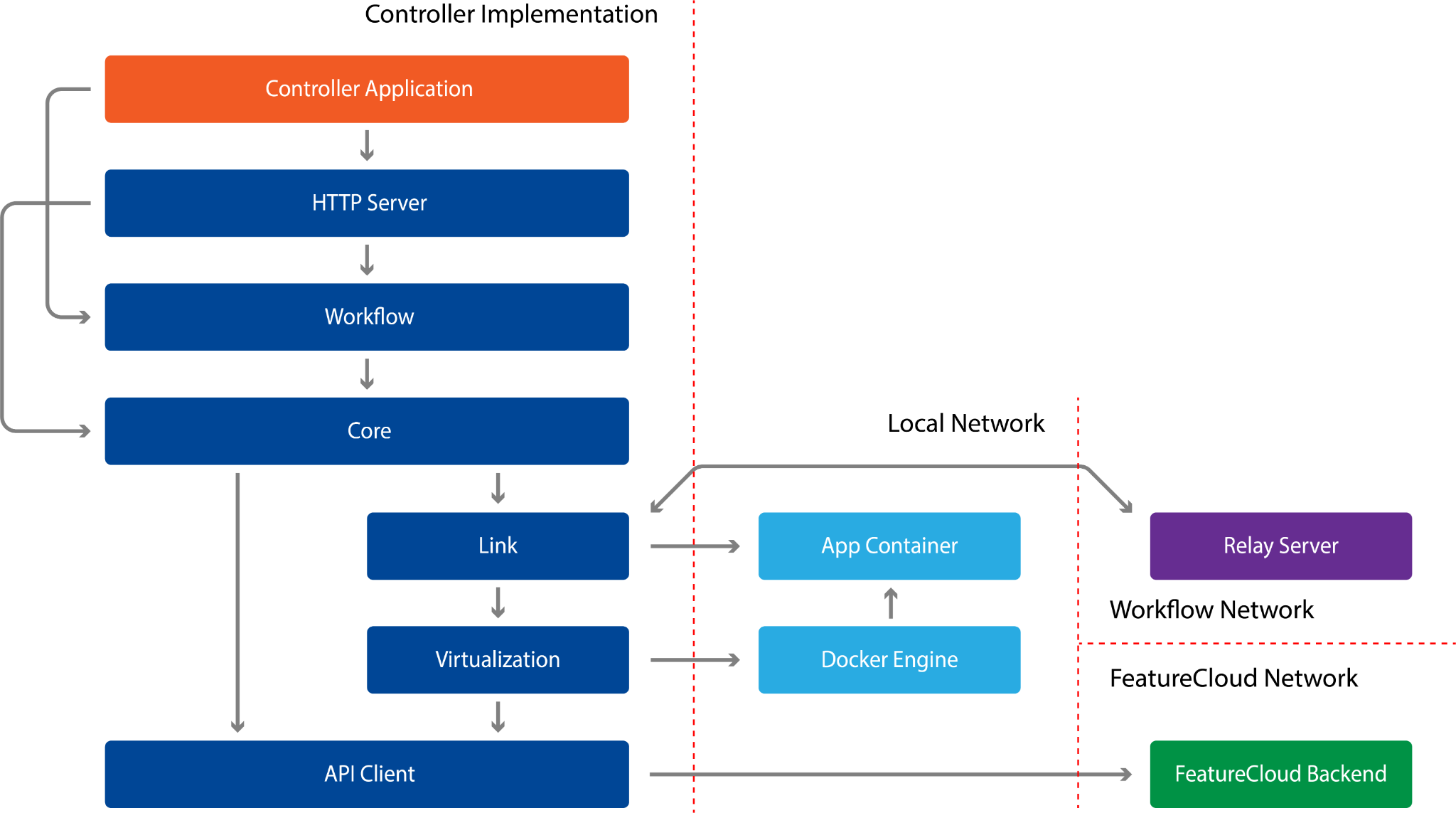
*

**Figure A1. Software architecture of the FeatureCloud Controller and its context.** The Controller uses a layered architecture preventing arbitrary access across layers by enforcing a partially ordered access hierarchy (left side). It communicates with the App Containers and the Docker Engine to orchestrate the local part of the workflow (middle). It receives instructions on which container to execute next from the FeatureCloud Backend (right side). The shared parameters are sent through the Relay Server (right side), which can reside in its own network to which all participants need to have access to. Alternatively, FeatureCloud provinces a Relay Server instance for convenience.

The software architecture has a layered structure, with a decreasing level of abstraction from top to bottom (see Figure A1).

The platform application layer is the main entry point responsible for reading configuration values (e.g. local database credentials, address of the global backend) and starting an HTTP server and polling routines. The HTTP server provides endpoints for the frontend to control workflow-related tasks, such as loading data into the first input volume and showing container logs. It also relays traffic to the app-specific frontends. The workflow layer offers abstract functions for the HTTP server and takes care of workflow management, such as setting up and attaching volumes, starting containers, shutting them down, reacting to updates from the global backend (by using the data layer through the core layer). The core layer provides an abstraction of the core business logic, especially app container management and functions for testing apps during development. The link layer handles communication between app containers and the relay server, translating raw byte-traffic from the relay server to HTTP-based traffic for the containers and vice versa. The controller acts as an HTTP client in this case and the app containers as HTTP servers. This way, active access by the app containers to the Internet can be avoided. The virtualization layer is a direct abstraction of Docker, which allows for replacing the virtualization technique in the future if needed for security or compatibility reasons.

## Relay Server

The relay server implements basic relay functionality for star-based federations of clients. It knows the role of each client (i.e. participant or coordinator) and treats their traffic accordingly. If data is received from a participant, it relays it to the coordinator. If it is received from the coordinator, it is broadcast to all clients. A relay server can handle multiple workflows at once. For that, it uses workflow-specific credentials chosen by the coordinator and automatically distributed to the participants by the global API. Like the controller, it is written in Go since it needs to efficiently handle large amounts of binary data, which Go is capable of.

## FeatureCloud Backend

The global backend mainly offers an HTTP API for controllers and the frontends. It is responsible for managing all necessary data related to projects, apps, users and data holders (sites). It is implemented in Django, a Python web framework that offers the functionality for this kind of task, particularly database abstraction, URL routing and web-related utilities (e.g. JSON serialization, HTTP abstraction).


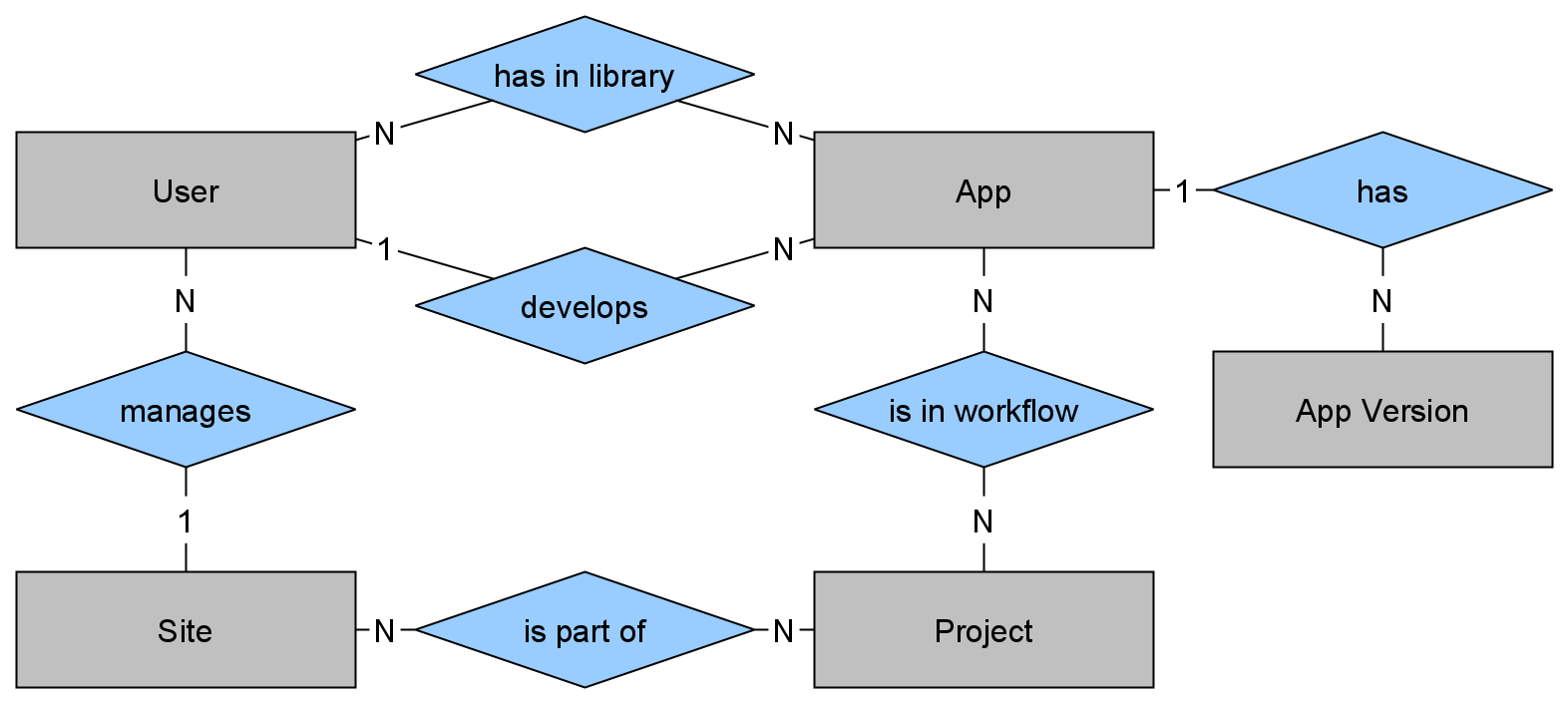


**Figure A2: E/R diagram of the data model in the backend.** Gray boxes represent entities, blue diamonds represent relationships.

The E/R diagram of the data model is shown in Figure A2. The global backend allows controlled access to instances of these entities.

*User.* Each user has an email address and a hashed and salted password to log in to the FeatureCloud frontend, which then queries the global backend. In practice, a user is either a developer who has apps linked to them through the ‘develops’ relation or an end-user. Both, developers and end-users, can add apps to their library (relation ‘has in library’) and manage a site (relation ‘manages’).

*Site.* Sites have necessary contact information and represent data holder locations, e.g. hospitals or academic research institutions. Each site needs to run a controller instance to participate in projects (relation ‘is part of’). When a site is part of a project, it can either assume the role of the coordinator or a participant.

*Project.* Projects encompass a workflow, descriptive information and a set of tokens allowing for joining a project. Tokens are not modeled explicitly. Instead, the ‘is part of’ table is used, which can have entries with a token string and where the related site is NULL. Once a site joins a project, this entry is linked accordingly and can no longer be used by anyone else.

*App.* Apps are AI applications that appear in the app store. They contain an image name, which needs to be used when pushing new versions of the app, an icon, a short and long description, tags, a category and a link to the source code. They are linked to a developer through the ‘develops’ relation and workflows they are part of through the ‘is in workflow’ relation.

*App Version.* New versions of apps are tracked automatically when pushing a new version via Docker by the developer and are linked to the respective app through the ‘has’ relation.

## Frontend

The frontend serves as a graphical user interface (GUI) for FeatureCloud users and developers. It is the only component FeatureCloud users directly interact with. It then calls the API of the controller or the global backend on behalf of the user, depending on the nature of the task. Since the frontend needs to be platform-independent, it has been implemented as a web application running inside a browser. This enforces a clear separation between GUI-related concerns and backend-related tasks by employing an HTTP-based API, as described earlier. Angular has been chosen as a web framework due to its high popularity, long-term support and extensive functionality.


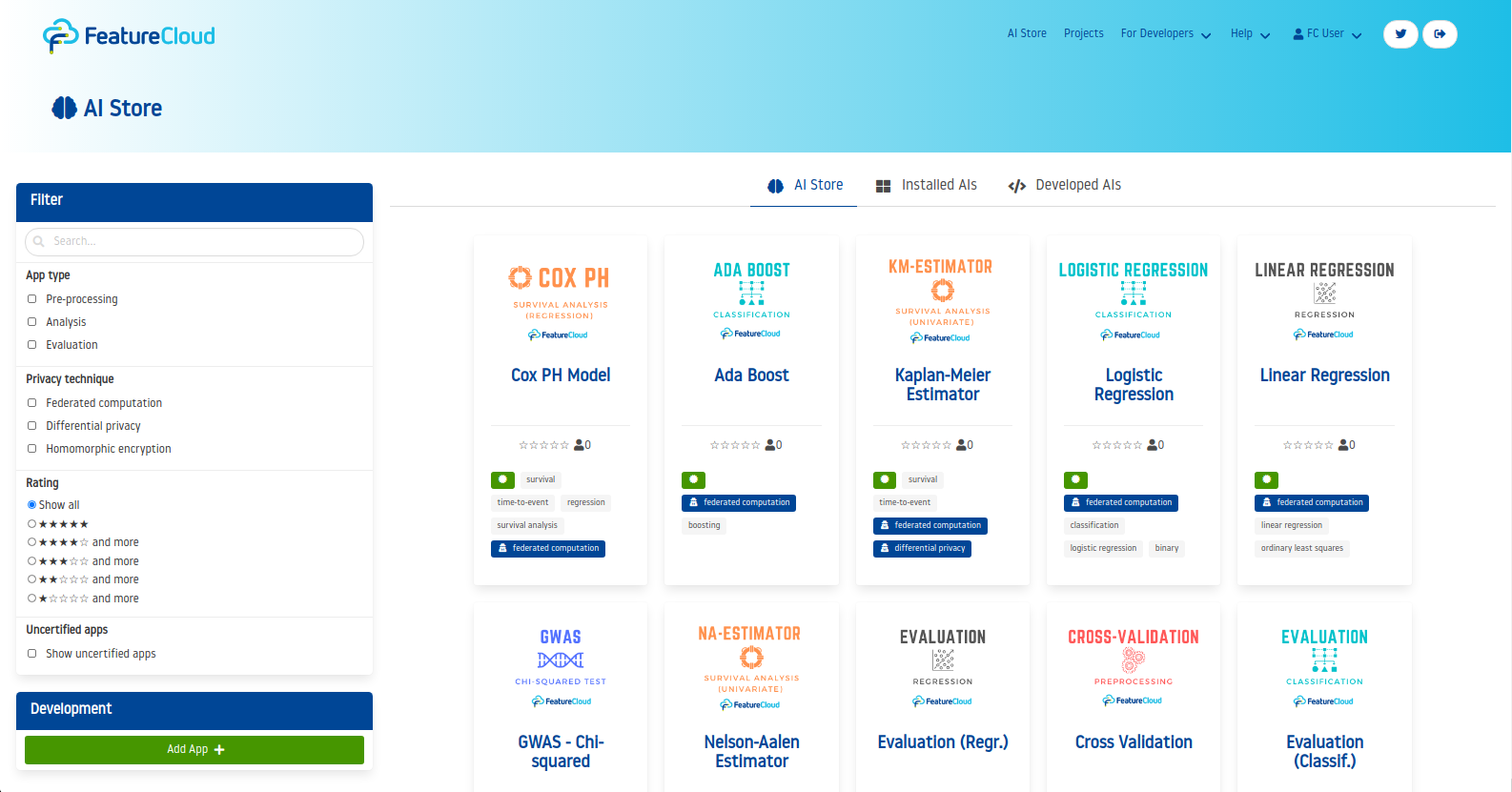


**Figure A3. The FeatureCloud AI Store.** The FeatureCloud AI Store provides an overview of all the federated learning apps. While end-users can find the appropriate apps for their desired federated workflow here, app developers can contribute their own apps here. Once certified by the FeatureCloud team, they will be publicly available for all FeatureCloud users.

The GUI is structured into the following sections (accessible through the menu): 1) Account management, 2) Site management, 3) App management, 4) Project management and 5) App testing, each divided into subsections again.

## Docker Registry

The AI Store server is connected to the global backend that serves as an authentication server and a Docker registry. It performs two main tasks: relay queries from the local Docker engines using the Docker registry API^^[[1]](#footnote-0)^^ and protecting images from unpermitted access, particularly restricting the pushing of images to the respective app developers. For that, the AI Store server provides endpoints to request a JWT token which is then attached automatically by the Docker CLI to authenticate consecutive actions. App developers must be FeatureCloud users and use their FeatureCloud credentials to log in. That way, the global backend acting as an auth server can check whether the user pushing an image is the corresponding app owner.

Like the controller, the relay server is written in Go for performance reasons. App images can be several GB large and pulling images is performed each time before a workflow step is executed, making performance a critical consideration.

# B. Certification process

After the development phase, apps can be published in the FeatureCloud AI Store. Developers need to fill out a form prompting all relevant information about the app, which is subsequently displayed to the end-users and utilized for the search and filter functions. After that, users can push their app to the Docker registry of the FeatureCloud platform. For end-users that explicitly enable uncertified apps, it is already usable and can be tested in the real world. For other end-users, we enforce a certification process to block malicious apps and maintain high privacy standards in the AI Store. To this end, the developer needs to provide necessary documentation and details about the implemented privacy mechanism. Furthermore, the source code of the app must be accessible so that the app can be exhaustively tested and vetted by the FeatureCloud team for possible privacy leaks or other security issues. When the certification process has been successfully completed by a member of the FeatureCloud consortium, according to a defined checklist (see Supplementary File B), the app will be displayed in the AI Store and can be used by all end-users. If the certification process was unsuccessful, the developer is notified and is requested to address the issues that have been raised. Upon each update of an application, a new certification procedure is triggered.


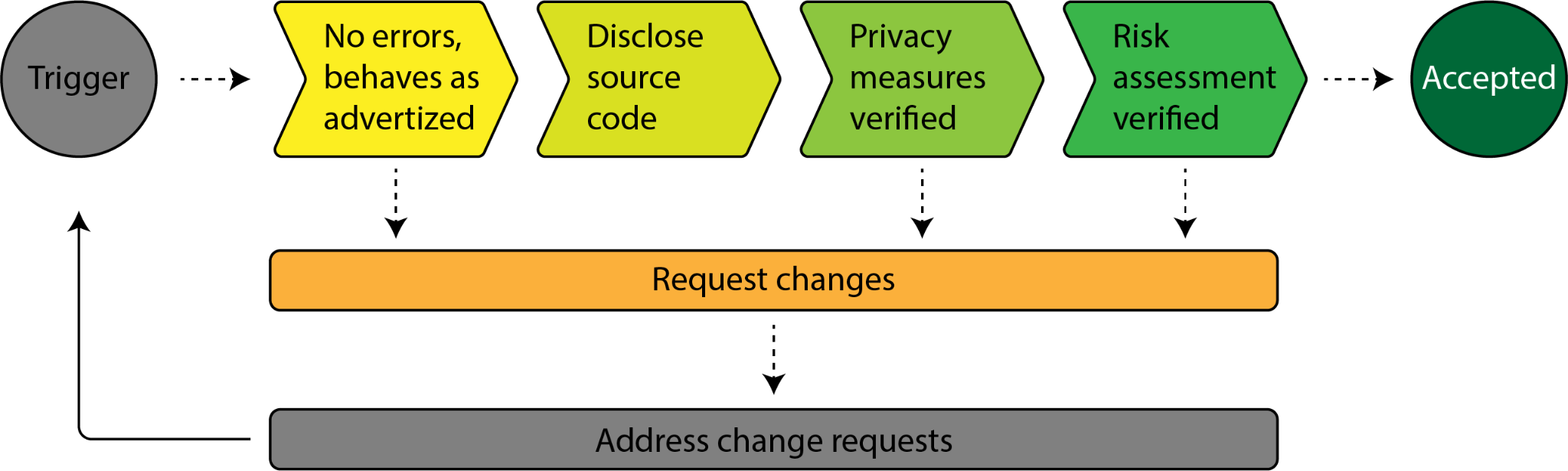


**Figure B1.** A new app and app updates need to undergo 4 stages during the certification process: Checking for errors, disclosing source code, verifying privacy measures, verifying risk assessment.

All apps in the AI Store are isolated to the highest possible degree, i.e. they do not have access to the filesystem or the internet. On top of that, the apps need to abide by our privacy standards. Herefore, they must undergo the following steps before being certified by a member of the FeatureCloud consortium and thus showing up in the public AI Store (see Figure B1).

1. The app must run without errors and provide the features advertised in the app description.
2. The complete source code must be disclosed so that the app image can be reproduced.
3. Privacy mechanisms that have been declared (e.g. DP, HE) must be implemented correctly and as stated in the app description.
4. The app developer has to elaborate on the data the app sends to the coordinator and assess the level of potential privacy leakage risks. In particular, no raw data must be handed over to the coordinator under any circumstances.

If these criteria are fulfilled, the app is built by the certifying party and pushed to the FeatureCloud AI Store. This way, it is ensured that no other image than the certified one will be executed on the systems of the end-users. The process will be repeated for each update of the app.

# C. Privacy-enhancing techniques

## Peer-to-peer messaging

Since the applied star-based architecture does not allow for sending data directly from one participant to another, a peer-to-peer (P2P) protocol has been implemented on top of this architecture using asymmetric encryption (see Figure C1).

Assuming *n* participants, each of them first creates a pair of public and private keys, and reveals its public key to all other participants through the relay server. This allows each participant to prepare data destined for a particular participant by encrypting it with the respective public key, without the relay server being able to read it. These public keys are distributed before any P2P communication occurs. The public keys need to be verified either by comparing a fingerprint through a different communication channel or by using keys certified by a common trusted authority, like in Transport Layer Security (TLS).


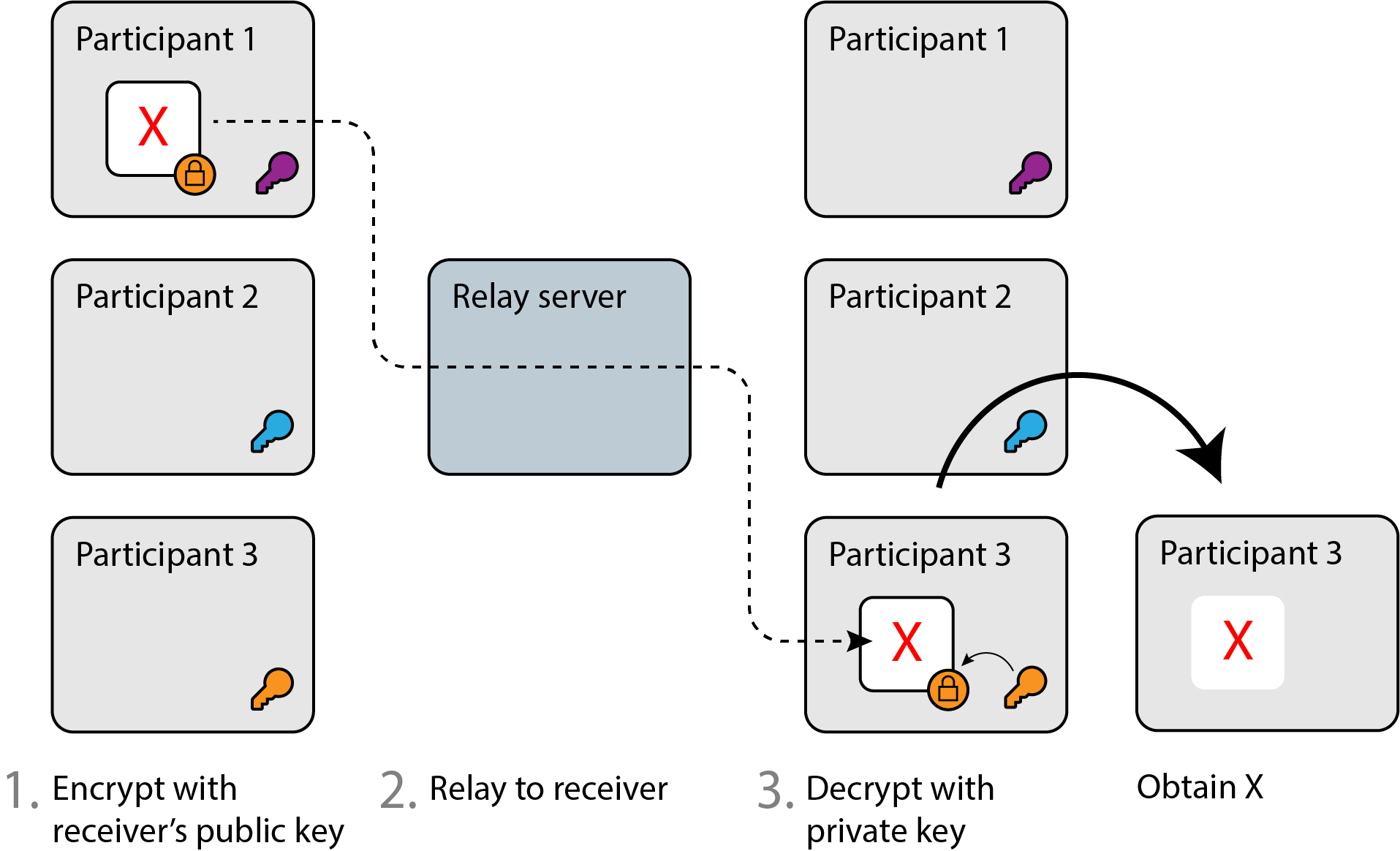


**Figure C1. Peer-to-peer communication built on top of star architecture.** In step 1, participant 1 encrypts value X using the public key of participant 3. In step 2, the relay server sends the encrypted value to participant 3, which decrypts it in step 3.

## Additive secret sharing

One of the crucial steps in FL is aggregating local models from multiple participants. This leads to an imbalance of required trust: while every participant will be able to see the aggregated model after an aggregation step, only the coordinator knows all individual models. To address this problem, an adapted additive secret sharing [(Cramer et al., 2015)](https://paperpile.com/c/mn3PQU/CzO8) technique has been implemented. Each participant splits its local model into *n* pieces or *secrets*, a masked model (M - r_1_ - … - r_n-1_), and the masks r_1_, …, r_n-1_ which are equally distributed random values. Those secrets are then distributed to the other parties. They, in turn, sum up all received pieces individually and send their sum to the coordinator, which can calculate the global sum and redistribute it to the other parties again (see Figure C2).


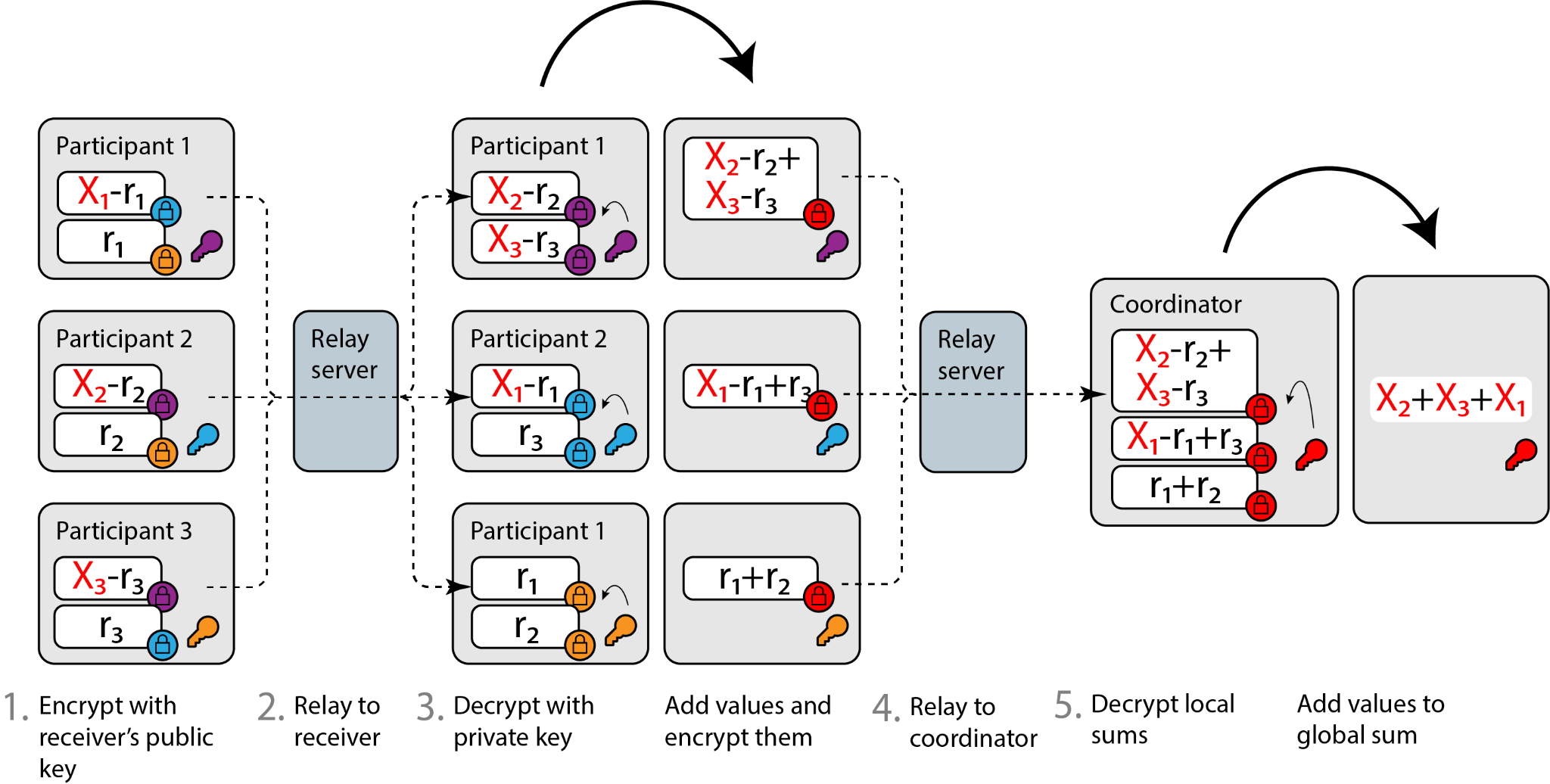


***Figure C2.*** *Additive secret sharing implemented in FeatureCloud. Step 1 shows how two secrets are created by each of the three participants for their values X_1_, X_2_ and X_3_. Step 2 distributes them according to the P2P protocol. In step 3, the received secrets are decrypted and summed up by each participant. Step 4 relays the local sums to the coordinator, which decrypts them in step 5 and calculates the global sum.*

When using this technique during training, at the beginning of each iteration, each participant first receives the global model (e.g., a randomly initialized neural network). Each participant then creates an updated model using its local data and masks the model with *n-1* different masks, one for each participant, and encrypts them with the respective participant’s public key. The masked model, together with the encrypted masks, is then sent back to the coordinator. The coordinator relays the encrypted masks to the participants who can decrypt their share of the masks and calculate the sum, which is then sent back to the coordinator. The coordinator finally sums up the masked models and the sums received from the participants to obtain the sum of local models. While providing enhanced privacy for each participant, it leads to an increase in network traffic, growing quadratic with the number of participants.

# D. Developers tools

The development of algorithms involves intensive testing and debugging. For rapid development, it is crucial that these testing and debugging cycles are as quick as possible. Therefore, FeatureCloud comes with a local test framework that enables app developers to instantly run their application on their machine without deploying it first. When using this functionality, one has to specify the number of participants, i.e. app instances to simulate, and a data directory for each instance containing the respective input data. When started, the FeatureCloud controller creates one container for each instance and connects them logically identically on the developer’s machine to a truly federated setup on different machines.

The API has deliberately been designed in an algorithm- and domain-agnostic way. This design leads to high flexibility but requires the app developer to implement all algorithm-specific functionality by themselves. To quickly introduce developers to the API and provide a convenient starting point for app development, FeatureCloud comes with a collection of easily extendable templates. This collection includes a minimal template with a demo Python implementation, stubs for all API calls and a blank demo frontend, and several federated implementations. They can be found on the FeatureCloud GitHub page^^[[2]](#footnote-1)^^.

To further facilitate development, FeatureCloud provides a pip package^^[[3]](#footnote-2)^^ with a CLI to create new apps from a template, start and stop the controller and run tests through the FeatureCloud system.

# E. Deep Learning Model

We used simple DL architecture with 2 fully connected layers and a tangent hyperbolic (tanh) activation function. For regularization, we added a dropout layer with a dropout rate of 0.3 and a batch-normalization layer with a momentum of 0.1, and an epsilon of 1e-5 after the first fully connected layer. For training, we chose an Adaptive Moment Estimation (Adam) optimizer with a learning rate of 1e-4 and a batch size of 128 and trained for 300 epochs.

# References

[Cramer, R., Damgard, I. B., & Nielsen, J. B. (2015). *Secure Multiparty Computation and Secret Sharing*. https://doi.org/](http://paperpile.com/b/mn3PQU/CzO8)[10.1017/cbo9781107337756](http://dx.doi.org/10.1017/cbo9781107337756)

[Rieke, N., Hancox, J., Li, W., Milletarì, F., Roth, H. R., Albarqouni, S., Bakas, S., Galtier, M. N., Landman, B. A., Maier-Hein, K., Ourselin, S., Sheller, M., Summers, R. M., Trask, A., Xu, D., Baust, M., & Cardoso, M. J. (2020). The future of digital health with federated learning. *NPJ Digital Medicine*, *3*, 119. https://doi.org/](http://paperpile.com/b/mn3PQU/p5HY)[10.1038/s41746-020-00323-1](http://dx.doi.org/10.1038/s41746-020-00323-1)

1. <https://docs.docker.com/registry/spec/api/> [↑](#footnote-ref-0)
2. <https://github.com/FeatureCloud> [↑](#footnote-ref-1)
3. <https://pypi.org/project/FeatureCloud/> [↑](#footnote-ref-2)
